# *Tmem79/Matt* is the matted mouse gene and is a predisposing gene for atopic dermatitis in human subjects

**DOI:** 10.1016/j.jaci.2013.08.046

**Published:** 2013-11

**Authors:** Sean P. Saunders, Christabelle S.M. Goh, Sara J. Brown, Colin N.A. Palmer, Rebecca M. Porter, Christian Cole, Linda E. Campbell, Marek Gierlinski, Geoffrey J. Barton, Georg Schneider, Allan Balmain, Alan R. Prescott, Stephan Weidinger, Hansjörg Baurecht, Michael Kabesch, Christian Gieger, Young-Ae Lee, Roger Tavendale, Somnath Mukhopadhyay, Stephen W. Turner, Vishnu B. Madhok, Frank M. Sullivan, Caroline Relton, John Burn, Simon Meggitt, Catherine H. Smith, Michael A. Allen, Jonathan N.W. N. Barker, Nick J. Reynolds, Heather J. Cordell, Alan D. Irvine, W.H. Irwin McLean, Aileen Sandilands, Padraic G. Fallon

**Affiliations:** a School of Medicine, Trinity College Dublin, Dublin, Ireland; b National Children's Research Centre, Our Lady's Children's Hospital, Dublin, Ireland; c Trinity Biomedical Sciences Institute, Trinity College Dublin, Dublin, Ireland; d Centre for Dermatology and Genetic Medicine, University of Dundee, Dundee, United Kingdom; e Biomedical Research Institute, University of Dundee, Dundee, United Kingdom; f Bioinformatics Research Group, University of Dundee, Dundee, United Kingdom; i Division of Cell Signalling and Immunology, University of Dundee, Dundee, United Kingdom; p Population Health Sciences, University of Dundee, Dundee, United Kingdom; g Bioinformatics Institute, A*STAR, Singapore; h Helen Diller Family Comprehensive Cancer Center, University of California, San Francisco, Calif; j Department of Dermatology, Allergy, and Venerology, University Hospital Schleswig-Holstein, Kiel, Germany; k Department of Pediatric Pneumology and Allergy, Children's Hospital Regensburg, Regensburg, Germany; l Institute of Genetic Epidemiology, Helmholtz Zentrum München, German Research Center for Environmental Health, Neuherberg, Germany; m Pediatric Pneumology and Immunology, Charité University Medicine Berlin and Max Delbrück Center for Molecular Medicine (MDC) Berlin-Buch, Berlin, Germany; n Academic Department of Paediatrics, Royal Alexandra Children's Hospital, Brighton and Sussex Medical School, Brighton, United Kingdom; o Department of Child Health, University of Aberdeen, Aberdeen, United Kingdom; q Institute of Genetic Medicine, Newcastle University, Newcastle, United Kingdom; r Department of Dermatology, Royal Victoria Infirmary, Newcastle upon Tyne, United Kingdom; s St John's Institute of Dermatology, King's College London, London, United Kingdom; t Institute of Cellular Medicine, Newcastle University, Newcastle, United Kingdom; u Department of Paediatric Dermatology, Our Lady's Children's Hospital, Crumlin, Dublin, Ireland

**Keywords:** Allergy, association, atopic dermatitis, atopy, eczema, filaggrin, flaky tail, *Matt*, mattrin, mouse, mutation, *Tmem79*, AD, Atopic dermatitis, DM, Double mutant, FLG, Filaggrin, HDM, House dust mite, hpf, High-power field, MAPEG, Membrane-associated proteins in eicosanoid and glutathione metabolism, OR, Odds ratio, SNP, Single nucleotide polymorphism, TEWL, Transepidermal water loss, WT, Wild-type

## Abstract

**Background:**

Atopic dermatitis (AD) is a major inflammatory condition of the skin caused by inherited skin barrier deficiency, with mutations in the filaggrin gene predisposing to development of AD. Support for barrier deficiency initiating AD came from flaky tail mice, which have a frameshift mutation in *Flg* and also carry an unknown gene, *matted*, causing a matted hair phenotype.

**Objective:**

We sought to identify the *matted* mutant gene in mice and further define whether mutations in the human gene were associated with AD.

**Methods:**

A mouse genetics approach was used to separate the *matted* and *Flg* mutations to produce congenic single-mutant strains for genetic and immunologic analysis. Next-generation sequencing was used to identify the *matted* gene. Five independently recruited AD case collections were analyzed to define associations between single nucleotide polymorphisms (SNPs) in the human gene and AD.

**Results:**

The matted phenotype in flaky tail mice is due to a mutation in the *Tmem79/Matt* gene, with no expression of the encoded protein mattrin in the skin of mutant mice. *Matt*^*ft*^ mice spontaneously have dermatitis and atopy caused by a defective skin barrier, with mutant mice having systemic sensitization after cutaneous challenge with house dust mite allergens. Meta-analysis of 4,245 AD cases and 10,558 population-matched control subjects showed that a missense SNP, rs6694514, in the human *MATT* gene has a small but significant association with AD.

**Conclusion:**

In mice mutations in *Matt* cause a defective skin barrier and spontaneous dermatitis and atopy. A common SNP in *MATT* has an association with AD in human subjects.

Atopic dermatitis (AD) is the most common diagnosis in dermatology, affecting approximately 1 in 5 children in the developed world,^1^ and is frequently associated with atopic asthma and a wide range of allergies.^2^ AD is a highly heritable complex trait; however, environmental influences also play a role in triggering the atopic diathesis.^3^ Genome-wide association studies in AD have identified several susceptibility loci^4-7^; however, the major and only functionally characterized genetic factor is the filaggrin gene *(FLG)*, which encodes the skin barrier protein filaggrin.^3^ Prevalent loss-of-function variants in *FLG* were identified as the cause of the single-gene disorder ichthyosis vulgaris (dry flaky skin).^8^ Soon thereafter, these variants were shown to be strongly associated with AD,^9^ with heterozygous odds ratios (ORs) of greater than 7 and homozygous ORs of greater than 150 in case-control studies in which both prevalent and rare variants were analyzed.^10^

The hypothesis that skin barrier deficiency in the context of *FLG* mutations is an initiator of AD was confirmed experimentally by using the flaky tail mouse mutant,^11^ which was shown to carry a frameshift mutation in the murine *Flg* gene.^12^ Flaky tail mice have a defective skin barrier, with increased percutaneous transfer of antigens and chemical haptens.^12-15^ The *ft* mutation arose spontaneously in 1958 in the progeny of crosses between heterogeneous stocks of mice with the recessive mutation *matted (ma)*, and these mutations are maintained as a double-mutant (DM) strain known as *maft*. The matted hair phenotype was used for many years as a surrogate marker for the *ft* mutation because, remarkably, the *ft* and *ma* mutations are closely linked on chromosome 3 in the mouse.^16^ The DM *maft* mice have been routinely used for studies of skin barrier–deficient AD in recent years.^13-15,17^ In mice and human subjects the *FLG* gene resides in the epidermal differentiation complex, a cluster of more than 70 genes encoding proteins involved in skin barrier formation and differentiation of stratified epithelia,^18^ including those within the hair follicle.^18-21^ We suspected the nearby *ma* gene might also be involved in epithelial barrier function, and in this study we set out to separate this allele from *Flg*^*ft*^ and identify the causative defect.

## Methods

### Isolation of the matted mouse strain

**Fig E1:**
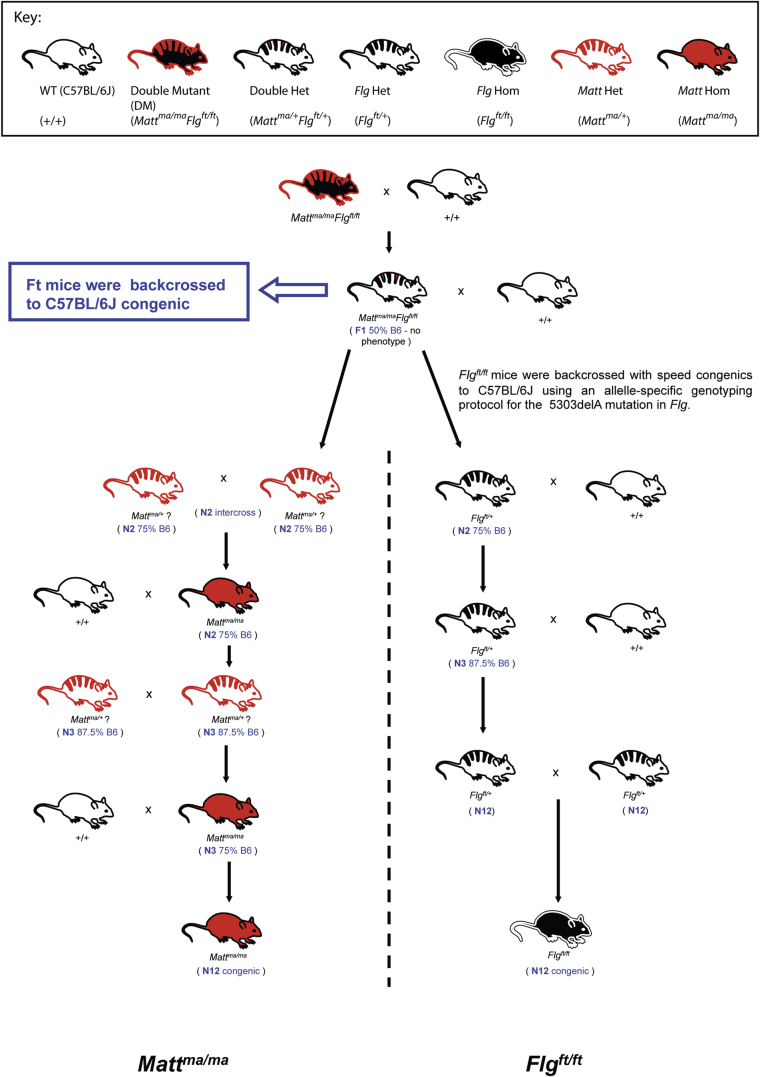


DM *Matt*^*ma/ma*^*Flg*^*ft/ft*^ mice were provided by Dr John P. Sundberg (Jackson Laboratory, Bar Harbor, Me).^12^ DM mice were crossed with C57BL/6J mice to generate *Matt*^*ma/+*^*Flg*^*ft/+*^ mice. The *Flg*^*ft*^ and *Matt*^*ma*^ mutations were separated and backcrossed to congenic C57BL/6J background in accordance with the breeding strategy outlined (see Fig E1 in this article's Online Repository at www.jacionline.org). C57BL/6J mice were used as wild-type (WT) control animals. B6.CBAGr-ma/J (JAX *Matt*^*ma/ma*^) mice were obtained from the Jackson Laboratory. Mice were housed in specific pathogen-free conditions, with irradiated diet and bedding and water *ad libitum*. All animal experiments were performed in compliance with Irish Department of Health and Children regulations and approved by Trinity College Dublin's BioResources Ethical Review Board.

### Gene mapping

Skin samples were obtained from neonatal mice, and DNA was extracted by using the DNA Purification Kit (Promega, Madison, Wis). Genomic DNA extracted from murine neonatal blood was amplified with the GoTaq Flexi DNA Polymerase kit (Promega). All samples were sequenced by using the ABI 3730 DNA Systems (Applied Biosystems, Foster City, Calif). Mapping primers were used to amplify and sequence murine chromosome 3 (see Table E1, *A*, in this article's Online Repository at www.jacionline.org). *Matted* genomic sequences were compared with C57BL/6J for regions of congenicity.

### Next-generation sequencing and bioinformatics

Three replicates from each sample (WT and *Matt*^*ma/ma*^) were submitted for next-generation sequencing. The replicates were run multiplexed on an Illumina GAIIx and HiSeq 2000 (Illumina, San Diego, Calif) by using v3 sequencing chemistry and the Roche 454 Titanium workflow (Roche, Mannheim, Germany). For further details on sequencing, bioinformatics, and single nucleotide polymorphism (SNP) and InDel methodologies, see the Methods section in this article's Online Repository at www.jacionline.org.

### Analysis and identification of murine *Tmem79/Matt* gene

Each of the 4 exons was amplified individually by using PCR with the following conditions for all exons: 1 cycle of 94°C for 5 minutes; 35 cycles of 94°C for 30 seconds, 54°C for 30 seconds, and 72°C for 1 minute; and a final extension at 72°C for 5 minutes.

### Semiquantitative RT-PCR

Mouse tissue samples were lysed with TissueLyser LT (Qiagen, Hilden, Germany), and RNA was extracted with the RNeasy kit (Qiagen). RNA was reverse transcribed with the ImProm-II Reverse Transcription System (Promega). An intron-spanning amplification was carried out on *Tmem79/Matt* across exons 3 and 4. *Krt14* was used as a loading control. RT-PCR primers used are shown in Table E1, *B*, in this article's Online Repository.

### Mouse genotyping

The 644-bp PCR product of the third *Tmem79/Matt* exon was digested with the restriction enzyme CviQI (New England Biolabs, Ipswich, Mass), and digested fragments were separated on agarose gel by using electrophoresis. Primers used are shown in Table E1, *A*. The *Flg*^*ft*^ mutation was genotyped, as previously described.^12^

### Immunoblotting

The epidermis was separated from the dermis of neonatal mice after immersion in 5 mmol/L EDTA at 50°C for 5 minutes, followed by cooling in ice-cold PBS. The separated epidermis was extracted in urea/Tris buffer containing protease inhibitor cocktail (Halt; Thermo Scientific, Erembodegem, Belguim) by means of homogenization. Protein samples were separated on SDS-polyacrylamide gels and transferred to polyvinylidene difluoride membranes (Millipore, Temecula, Calif), which were probed with rabbit polyclonal antibodies specific for human mattrin (TMEM79; Novus Biologicals, Littleton, Colo). Primary antibodies were detected by means of incubation with a horseradish peroxidase–conjugated goat anti-rabbit secondary antibody (Dako, Stockport, United Kingdom). Immunolabeled proteins were visualized by using chemiluminescence with the ECL detection system (Millipore).

### Immunofluorescence and Nile Red staining

Dorsal murine sections were obtained from 4-day-old neonatal mice and snap-frozen immediately. All frozen samples were then cryosectioned at 4 to 5 μm and stored at 80°C until use. After drying, sections were fixed in 50% methanol acetone, washed in PBS, and incubated with primary antibodies. Nuclei were stained with 4′-6-diamidino-2-phenylindole (Sigma-Aldrich, St Louis, Mo), and slides were fixed in Hydromount (National Diagnostics, Atlanta, Ga). Primary antibodies used in whole mounts were as follows: polyclonal TMEM79 (TMEM79; Novus Biologicals) and monoclonal CK-13 against cytokeratin 17 (Sigma-Aldrich). Frozen mouse skin sections were stained with Nile Red and 6-diamidino-2-phenylindole. Images were collected with a Zeiss LSM700 confocal microscope (Zeiss, Oberkochen, Germany).

### Quantitative PCR of human cDNA tissue array

Expression of *TMEM79/MATT* cDNA was quantified across a multiple-tissue panel containing cDNA from 48 different human tissues (Origene Technologies, Rockville, Md). This was done with the TaqMan Gene Expression Assay, C_25986870_10 (Life Technologies, Carlsbad, Calif), which spans exons 3 to 4 to result in amplicon length of 65 bp in cDNA sequence. The standard used in this assay was a commercially available plasmid containing the full-length WT *TMEM79/MATT* (Origene Technologies). A standard curve was calculated by using the formulae provided by Applied Biosystems.

### Phenotypic scoring

The severity of inflammation and AD-like pathology was scored by using the macroscopic diagnostic criteria described for the skin inflammation seen in the Nc/Nga mouse model of AD.^22^ Briefly, a scoring system (0, none; 1, mild; 2, moderate; and 3, severe) was applied to the symptoms of pruritus, edema, erosion, scaling, and erythema. Pruritus (scratch behavior) was observed for 5 minutes. Total scores for each mouse were calculated from the sum of individual scores.

### Cutaneous challenge with house dust mite antigen

Mice were challenged with house dust mite (HDM) to induce allergic skin sensitization, as previously described.^13^ Briefly, HDM (Greer Laboratories, Lenoir, NC) was topically applied to intact skin of WT and *Matt*^*ma*^ mice by using Finn chamber patch tests (SmartPractice, Phoenix, Ariz) and secured with Scanpor tape (Bio-Diagnostics, Worcester, United Kingdom). The regimen involved five 1-week exposures to the allergen patch separated by 2-week intervals. Hair was clipped 24 hours before application of HDM that was prepared in endotoxin-free Dulbecco PBS and vehicle to a concentration of 1 mg/mL. Dulbecco PBS was used as the control. Mice were assessed 24 hours after the final HDM challenge.

### ELISA

Serum levels of HDM-specific IgE, IgG_1_, and IgG_2a_ were detected by means of direct ELISA. Total serum IgE levels were measured with a sandwich ELISA, according to the manufacturer's instructions (BD PharMingen, San Jose, Calif).

### Histology

Dorsal skin sections were removed and fixed in 10% formal saline. Paraffin-embedded sections were stained with hematoxylin and eosin and examined in blinded fashion by 2 observers independently. To quantify dermal cell numbers, we used a previously described scoring system.^12^ The total number of cells per high-power field (hpf) of view were counted on 20 hpfs per mouse. For skin sections, an arbitrary histologic scoring system was used to quantify acanthosis and hyperkeratosis. Acanthosis scoring was based on the magnitude of epidermal hyperplasia, and hyperkeratosis scoring was based on the magnitude of stratum corneum thickening, with scores (0, basal; 1, mild; 2, moderate; 3, marked; 4, very marked; and 5, extreme pathology) for each parameter based on measurements per hpf in sections.

### Measurement of transepidermal water loss

A Courage and Khazaka Tewameter TM210 (Enviroderm, Evesham, United Kingdom) was used for measurement of transepidermal water loss (TEWL), which was measured on the dorsal flank of mice 24 hours after hair clipping. TEWL was recorded at an ambient temperature of 19°C to 21°C and humidity of 50% ± 5%. In mice sensitized to HDM, TEWL was measured in PBS- and HDM-treated mice before and after the HDM challenge regimen.

### Electron microscopy

For scanning electron microscopy, hair fibers were removed from mice and fixed in cacodylate-buffered glutaraldehyde. Samples were prepared for scanning electron microscopy by using established procedures^11^ and examined in a Zeiss Supra scanning electron microscope.

### Statistical analysis of mouse studies

GraphPad Prism software (GraphPad Software, La Jolla, Calif) was used for data analysis. Differences between groups were determined by using the Student *t* test and 2-way ANOVA. Results are presented as means ± SEMs. Differences, indicated as 2-tailed *P* values, were considered significant at a *P* value of less than .05.

### SNP identification in human subjects

Human samples from England, Scotland, and Ireland were obtained with consent from patients and Institutional Ethics Committee approval complying with the principles of the Declaration of Helsinki. Human *MATT* was sequenced by using primers listed in Table E1, *C*, in this article's Online Repository at www.jacionline.org. Conditions for all exons except exon 2.2 were as follows: 1 cycle of 94°C for 5 minutes; 35 cycles of 94°C for 30 seconds, 54°C for 30 seconds, and 72°C for 1 minute; and a final extension at 72°C for 5 minutes. An annealing temperature of 58°C was used for exon 2.2. SNP rs6684514 (see Table E2 in this article's Online Repository at www.jacionline.org) was genotyped across the case-control study populations by using the TaqMan allelic discrimination genotyping assay (C_25986870_10, Life Technologies Corporation).

### Case-control studies

Five independently recruited AD case collections were compared with ethnically matched population control subjects (see the Methods section in this article's Online Repository). Case definitions are described in the Methods section in this article's Online Repository, and demographic data relating to these AD case collections and control subjects are summarized in Table E3 in this article's Online Repository at www.jacionline.org.

### Genotype imputation

For *in silico* replication, we used the 2 German AD case-control selections (see Table E3), which had been genotyped on Illumina 300k and Affymetrix 6.0p (Affymetrix, Santa Clara, Calif), respectively, and imputed based on the 1000 Genomes database (phase I integrated variant set release, March 2012). Previous imputation data sets were curated, and samples with a greater than 5% missing rate, excess of heterozygosity (±5 SDs away from the sample mean), and unexpected relatedness (PI_HAT>0.1875, halfway between expected IBD for third- and second-degree relatives) were excluded. Further outliers from the multidimensional scaling of the pairwise IBS matrix were removed. Imputation was carried out with SHAPEIT and IMPUTE2. The SNP rs6684514 investigated *in silico* had an imputation quality of 0.996 (proper info) and a CR of 0.997.

### Statistical genetics

Case-control comparisons for each case collection and population control subjects were performed by using logistic regression analysis in Stata 12.0 (StataCorp, College Station, Tex). Meta-analysis (under fixed- and random-effects models) of the resulting log ORs and their SEs was performed with the “metan” function in Stata 12.0.

## Results

### Identification of *Matt* as the gene mutation in *matted* mice

**Fig E2:**
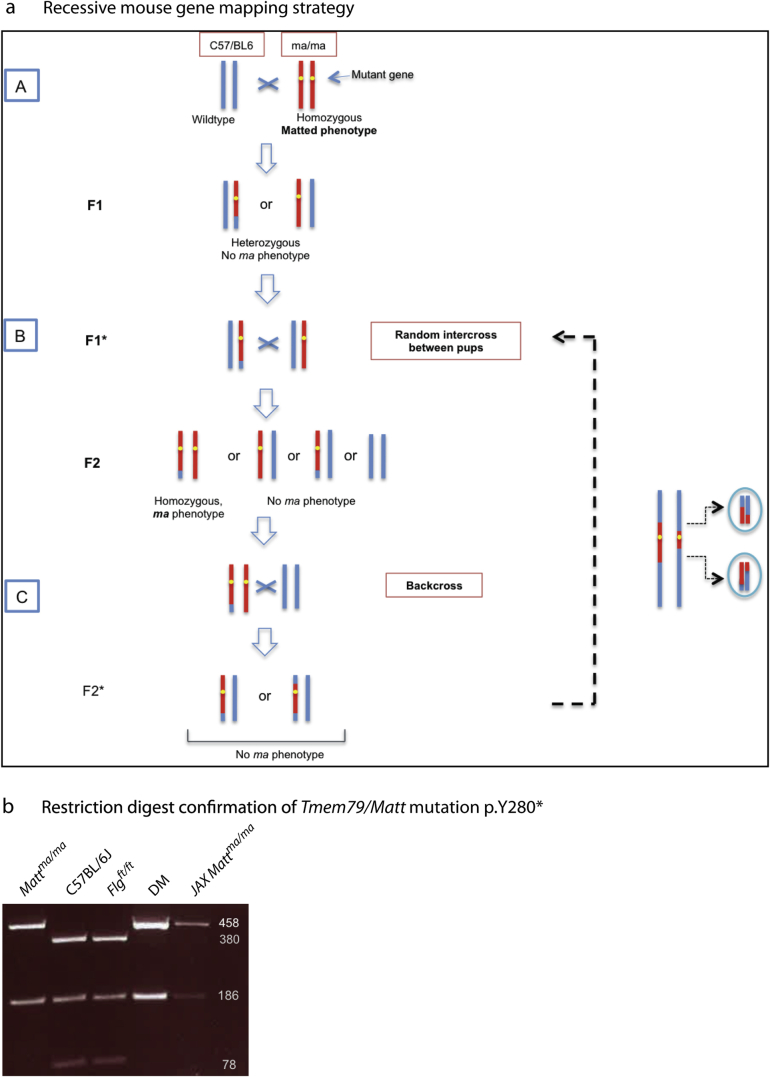


**Fig 1:**
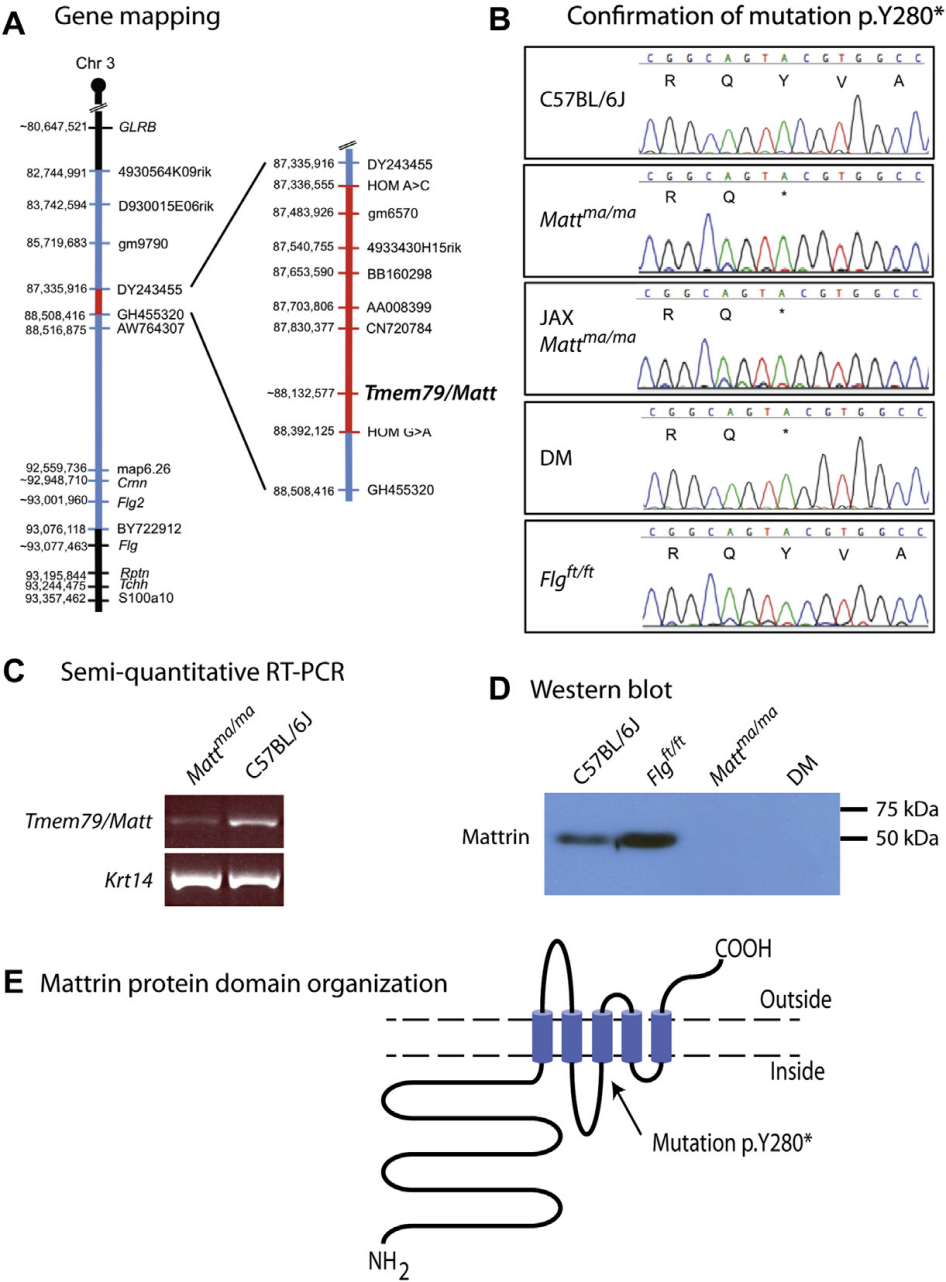
Identification of *Tmem79/Matt* as the *matted* gene. **A,** A region of homozygous *ma/ma* on chromosome 3 indicated by regions in *red* and heterozygous regions in *blue*, with the homozygous C57BL6/J genome shown in *black*. **B,** The *ma* mutation in *Tmem79/Matt* identified by means of epidermal transcriptome sequencing was confirmed by using Sanger sequencing, causing a C to G substitution at position 88,136,485 on chromosome 3. This predicts the protein change p.Y280X in the *Tmem79* (mattrin) protein. The mutant allele was renamed *Matt*^*ma*^. **C,** Semiquantitative RT-PCR of epidermal mRNA showed a reduction in expression of *Matt* in *Matt*^*ma/ma*^ animals. **D,** Mattrin detected in immunoblotting of skin from WT and *Flg*^*ft/ft*^ mice but no protein expression in *Matt*^*ma/ma*^ and DM mice. **E,** Bioinformatics shows mattrin consists of 5 transmembrane helices with a long intracellular N-terminus and short extracellular C-terminus.

To separate the 2 mutations in the DM *maft* mice, we used the backcross-intercross protocol outlined in Fig E1. Mice with the *Flg*^*ft*^ mutant allele (*Flg* c.5303delA) were genotyped, as previously described.^12^ Progeny from putative heterozygous intercrosses were checked for the recessive matted hair phenotype to identify *ma/ma* homozygotes. By this means, we separated both alleles. Because SNP analysis showed that the initial DM *maft* mouse was maintained on a mixed-strain background, we generated single-mutant N12 mice congenic on the C57BL/6J background. By using this backcross-intercross strategy to generate congenic *ma/ma* homozygous mice (see Fig E1), the recessive disease–causing locus should be ultimately visible as a small area of homozygosity flanked by 2 unequal heterozygous regions (see Fig E2, *A*, in this article's Online Repository at www.jacionline.org). To map this, we sequenced several short PCR fragments across the region from C57BL/6J congenic *ma/ma* homozygotes (see Table E1, *A*) to identify SNPs. By this means, a 1.05-Mb critical interval was defined (Fig 1, *A*). In parallel, we used epidermal transcriptomic sequencing to compare WT C57BL/6J and congenic *ma/ma* homozygotes. A nonsense mutation, p.Y280*, in the *Tmem79* gene, located within the interval (Fig 1, *A*), was identified in the RNAseq data (not shown). The mutation in *Tmem79* was confirmed by using conventional sequencing of genomic PCR products (Fig 1, *B*) and restriction digests (see Fig E2, *B*). This mutant transcript was expressed at approximately 30% of the WT levels in *ma/ma* epidermis (Fig 1, *C*). By using a commercially available antibody against the C-terminus of the encoded protein, a single band of approximately 49 kDa was seen in epidermal extracts from WT and congenic *Flg*^*ft/ft*^ mice, with the protein absent in congenic *ma/ma* and DM mice (Fig 1, *D*), indicating the presence of a truncated protein or an absence of protein in these mice. Interestingly, epidermal extracts from *Flg*^*ft/ft*^ mice had increased expression of protein relative to WT extracts. *Tmem79* was renamed *Matt*, encoding the protein mattrin; similarly, the human ortholog was renamed *MATT* (approved by the Genome Nomenclature Committee). By convention, after identification of the gene, the *ma* mutant allele was redesignated as *Matt*^*ma*^.

### Mattrin is expressed in mouse and human skin

**Fig E4:**
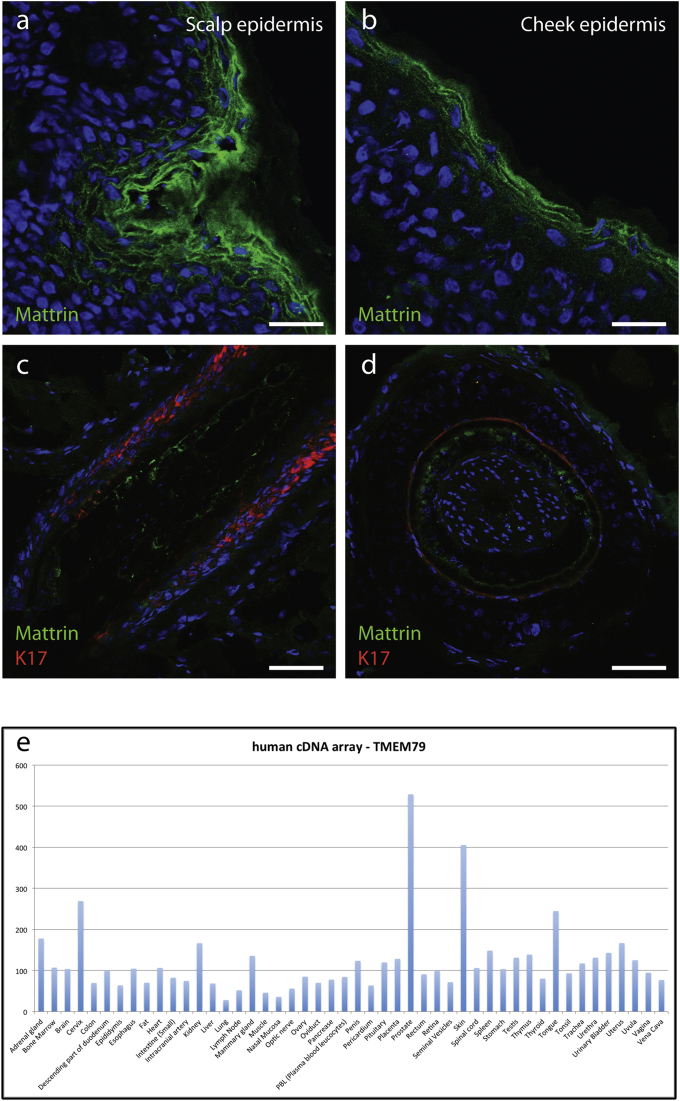


**Fig 2:**
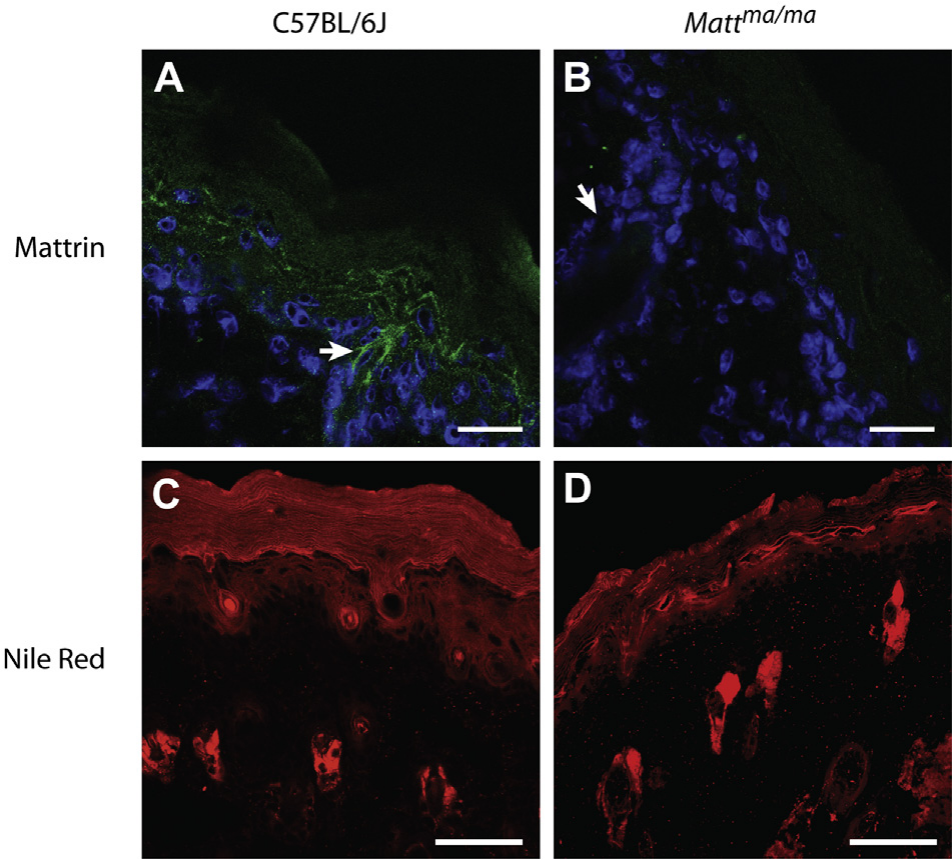
Mattrin expression and disorganized lipid morphology in the epidermis of *Matt*^*ma*^ mice. **A** and **B,** Immunohistochemistry detection of mattrin expression confined to the epidermal granular layer of WT (Fig 2, *A*) but not *Matt*^*ma/ma*^ (Fig 2, *B*) animals, including hair follicles *(arrow)*. *Scale bar* = 20 μm. **C** and **D,** Nile Red staining of lipids in the epidermis of WT (Fig 2, *C*) and *Matt*^*ma/ma*^ (Fig 2, *D*) mice revealed uneven and highly disorganized cornified cell envelopes in the stratum corneum of *Matt*^*ma/ma*^ mice. *Scale bar* = 50 μm.

*Matt* is a compact 5-exon gene on mouse 3qF1 spanning 5781 bp of genomic DNA, encoding a 391-amino-acid protein with a calculated molecular weight of 43.5 kDa. The human ortholog, *MATT*, which is syntenic on 1q23.1, has an identical organization. Mattrin is a transmembrane domain protein with no previously ascribed function (Fig 1, *E*). Bioinformatics analysis predicts a long internal N-terminal domain, 5 transmembrane domains, and a short C-terminus (Fig 1, *E*, and see Fig E3 in this article's Online Repository at www.jacionline.org). The mutation p.Y280* occurs before the third transmembrane domain (Fig 1, *E*). Immunohistochemistry revealed that mattrin was present in epidermal granular layer keratinocytes in WT mice (Fig 2, *A*) but was absent from *Matt*^*ma/ma*^ animals (Fig 2, *B*). Similarly, the protein was observed in granular layer cells in human epidermis (see Fig E4, *A* and *B*, in this article's Online Repository at www.jacionline.org) and was also found in the hair follicles of mice (Fig 2, *A*) and human subjects (see Fig E4, *C* and *D*). In addition, the mRNA is widely expressed in human tissues and, consistent with immunohistochemistry, is strongly expressed in skin (see Fig E4, *E*).

Expression quantitative trait locus analysis^23^ showed that *Matt* is in a network of proteins expressed late in epidermal differentiation, with an expression quantitative trait locus profile closely matching that of *Rhbg* (transporter protein) and *Rab25* (membrane trafficking, data not shown). Mattrin shows distant sequence homology to the Membrane-Associated Proteins in Eicosanoid and Glutathione metabolism (MAPEG) protein family (see Fig E3),^24^ members of which have roles in lipid catabolism.^25^ Immunohistochemistry with the lipophilic dye Nile Red revealed highly organized stacks of cornified cell envelopes in the stratum corneum of WT animals (Fig 2, *C*) that were highly disorganized in *Matt*^*ma/ma*^ mice, with discontinuous, uneven, and highly disorganized cornified cell envelopes (Fig 2, *D*).

### *Matt*^*ma/ma*^ mice have spontaneous dermatitis

**Fig E5:**
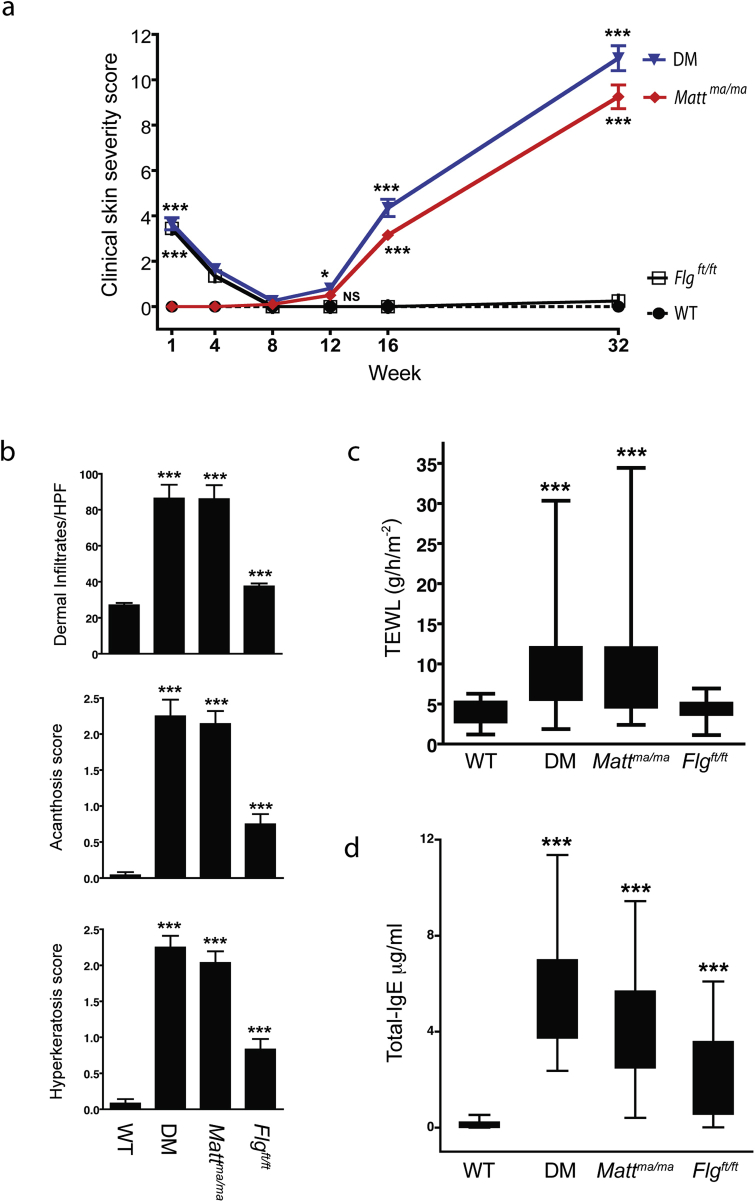


**Fig E6:**
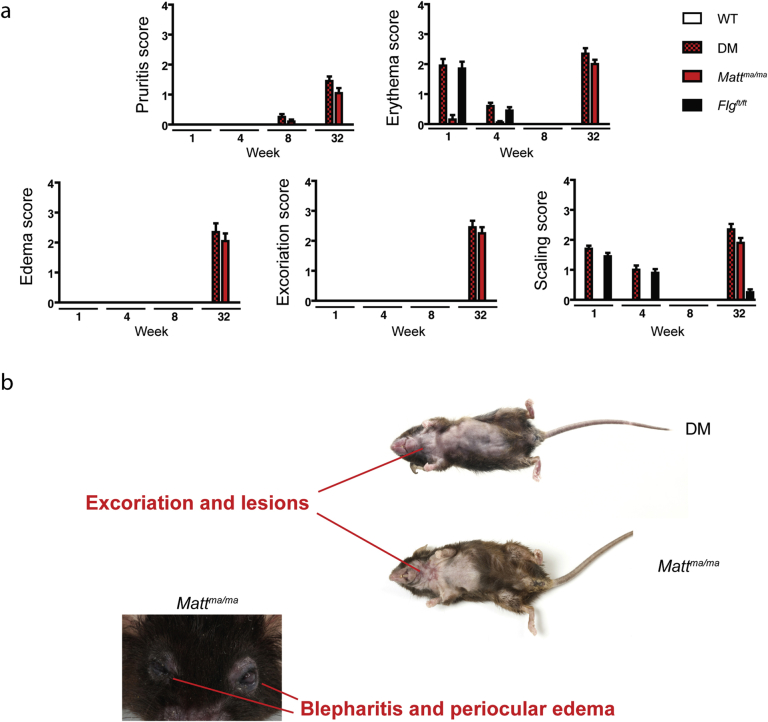


**Fig 3:**
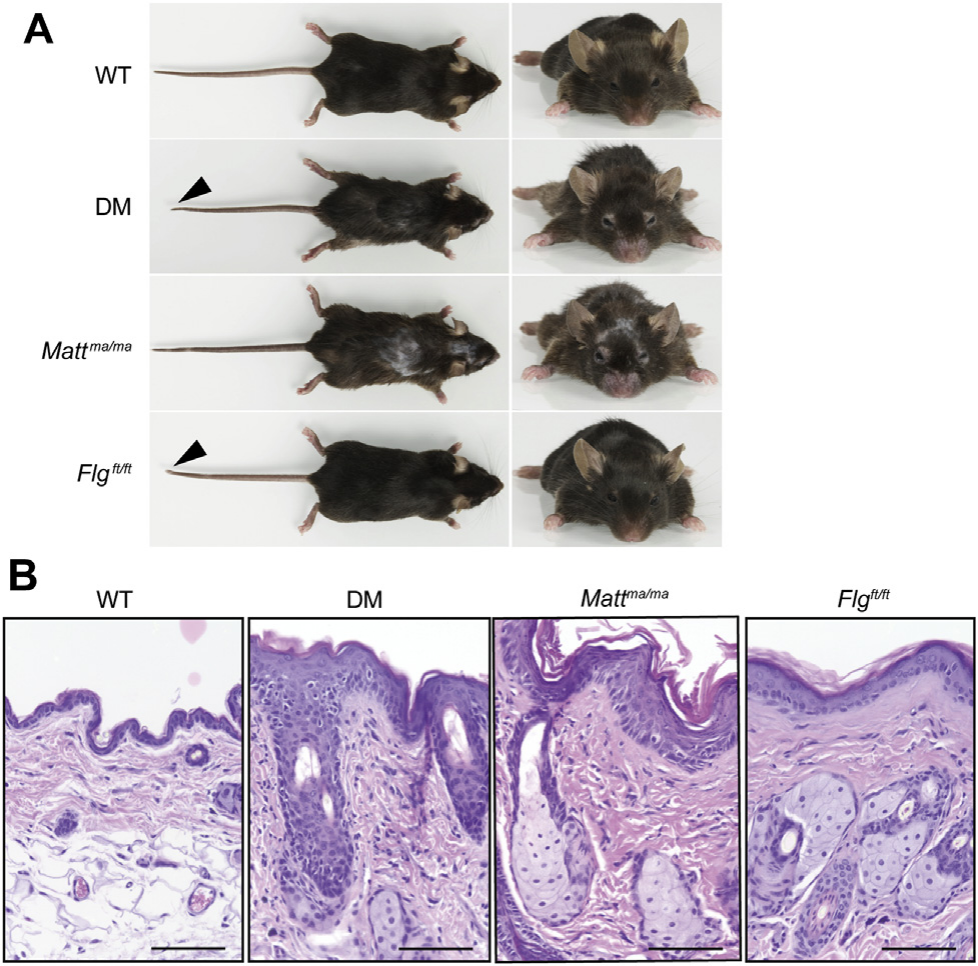
*Matt*^*ma*^ mice are phenotypically distinct from *Flg*^*ft/ft*^ mice, with histopathology demonstrating enhanced cutaneous inflammation at 32 weeks. **A,** Gross phenotype of WT, DM, *Matt*^*ma/ma*^, and *Flg*^*ft/ft*^ mice. *Flg*^*ft/ft*^ mice are indistinguishable from WT mice, apart from the stubbed tail and shortened ear pinnae, which are phenotypic features shared with the DM mouse. All mice are age-matched homozygous males. **B,** Representative skin biopsy specimens from WT, DM, *Matt*^*ma/ma*^, and *Flg*^*ft/ft*^ mice at 32 weeks, showing markedly increased cutaneous inflammation in DM and *Matt*^*ma/ma*^ mice in comparison with that seen in WT and *Flg*^*ft/ft*^ mice. *Scale bar* = 50 μm.

Gross examination of adult *Flg*^*ft/ft*^ and *Matt*^*ma/ma*^ mice demonstrates that the 2 strains differ considerably from each other (Fig 3, *A*). Macroscopic clinical scoring^22^ demonstrated *Matt*^*ma/ma*^ and DM mice spontaneously having progressive dermatitis-like skin inflammation, which did not occur in adult *Flg*^*ft/ft*^ animals (see Fig E5, *A*, in this article's Online Repository at www.jacionline.org). Although all *Matt*^*ma/ma*^ and DM mice had marked skin inflammation, there was a broad spectrum of pathology, with some animals exhibiting profound lesions and excoriation and occasional blepharitis and eyelid dermatitis (see Fig E6, *B*, in this article's Online Repository at www.jacionline.org). Interestingly, both neonatal *Flg*^*ft/ft*^ and DM mice have significant ichthyosis (*P* < .001) compared with WT mice, but *Matt*^*ma/ma*^ mice did not, indicating the postnatal importance of filaggrin (see Fig E5, *A*, and E6, *A*).

**Fig E7:**
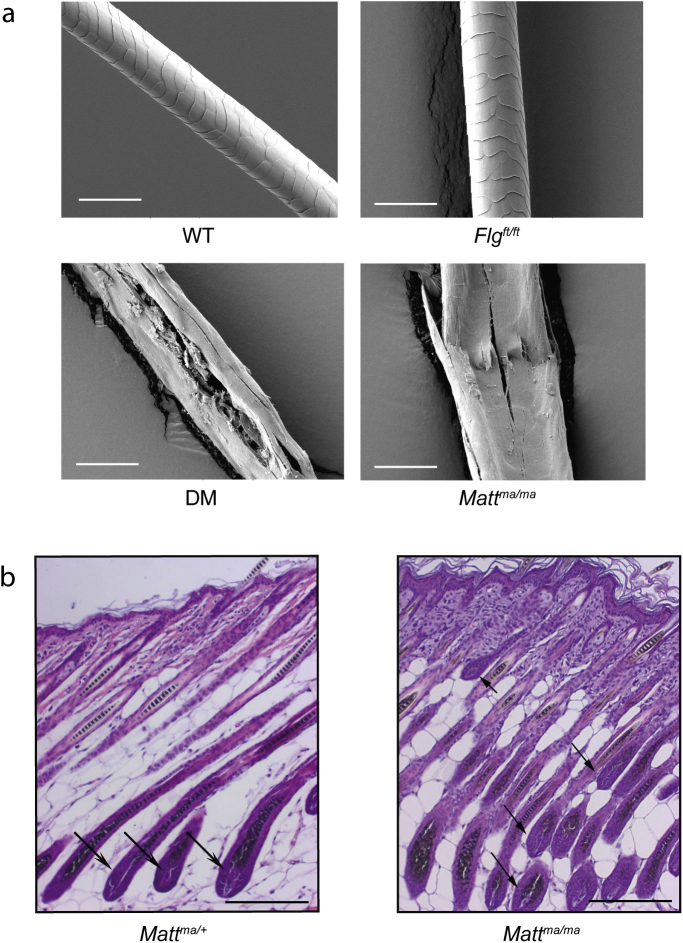


With respect to the matted hair phenotype,^11,26^ both *Matt*^*ma/ma*^ and DM animals had the keratinization defect, with hairs forming clumps^11,26^ and hair breakage and occasional alopecia evident at 32 weeks (Fig 3, *A*, and see Fig E6, *B*). Scanning electron microscopy confirmed *Matt*^*ma/ma*^ and DM mice had fragile hairs prone to longitudinal splitting and breakage with defective cuticle morphology (see Fig E7, *A*, in this article's Online Repository at www.jacionline.org). Furthermore, *Matt*^*ma/ma*^ mice had distorted hair follicle morphogenesis (see Fig E7, *B*).

Skin histology (Fig 3, *B*) showed that *Matt*^*ma/ma*^ and DM mice have marked acanthosis (*P* < .001) and prominent orthokeratosis (*P* < .001) with dermal inflammatory infiltrates (*P* < .001; see Fig E5, *B*). In contrast, *Flg*^*ft/ft*^ mice had occasional foci of acanthosis, mild diffuse orthokeratosis, and increased dermal cell infiltration relative to WT mice (*P* < .001). However, the phenotype in *Flg*^*ft/ft*^ mice was subclinical, with no overt dermatitis (Fig 3, *A*, and see Fig E5, *A*).

### *Matt*^*ma/ma*^ mutant mice are atopic and have a defective skin barrier

All mutant strains spontaneously had increased IgE levels relative to WT mice, which was markedly more pronounced in *Matt*^*ma/ma*^ and DM animals relative to *Flg*^*ft/ft*^ mice (*P* < .001; see Fig E5, *D*). Because the severity of skin inflammation in AD parallels barrier permeability,^27,28^ TEWL was analyzed to quantify skin barrier dysregulation in mouse strains. TEWL was significantly increased in adult *Matt*^*ma/ma*^ and DM mice (*P* < .001; see Fig E5, *C*). In contrast, TEWL was unaltered in *Flg*^*ft/ft*^ mice relative to that seen in WT animals (see Fig E5, *C*).

**Fig 4:**
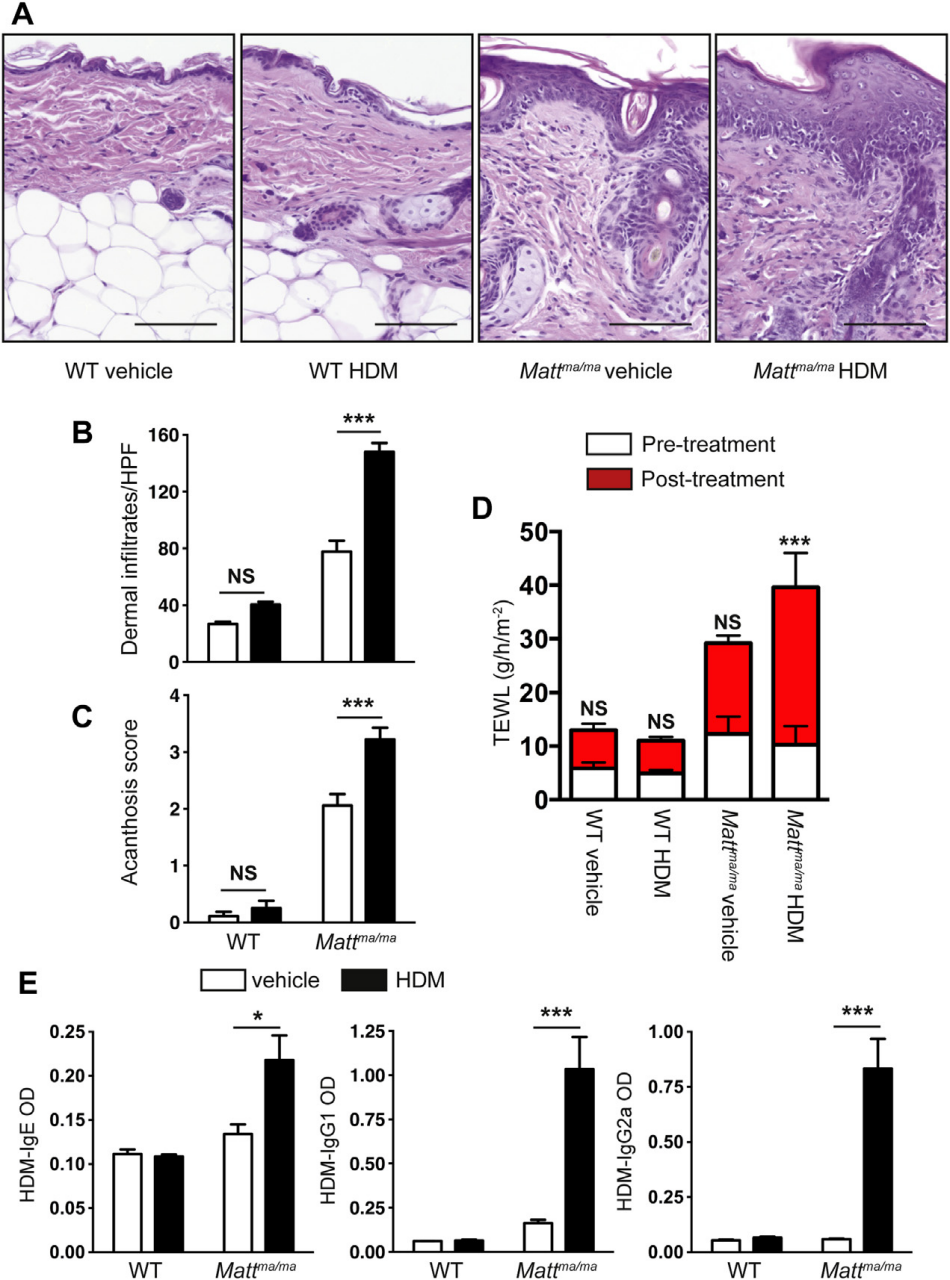
*Matt*^*ma*^ mice have exacerbated AD-like inflammation and HDM-specific responses after allergen challenge to intact skin. **A,** Representative skin biopsy specimens from age-matched WT and *Matt*^*ma/ma*^ mice treated with HDM or vehicle, with increased inflammation in HDM-treated *Matt*^*ma/ma*^ mice relative to that seen in HDM-treated WT and vehicle-treated *Matt*^*ma/ma*^ mice. *Scale bar* = 50 μm. **B,** HDM treatment induces increased dermal cell infiltration in *Matt*^*ma/ma*^ mice relative to that seen in HDM-treated WT and vehicle-treated *Matt*^*ma/ma*^ mice. **C,** Acanthosis is increased in HDM-treated *Matt*^*ma/ma*^ mice relative to that seen in HDM-treated WT and vehicle-treated *Matt*^*ma/ma*^ mice. **D,** Epicutaneous HDM treatment results in significantly increased TEWL in *Matt*^*ma/ma*^ mice relative to that seen in HDM-treated WT mice. **E,** HDM-treated *Matt*^*ma/ma*^ mice have increased HDM-specific serum IgE, IgG_1_, and IgG_2_ levels relative to those seen in HDM-treated WT mice. Cell numbers and acanthosis were scored on 15 to 20 hpfs (×1000 magnification) on hematoxylin and eosin–stained sections from WT, DM, *Matt*^*ma/ma*^, and *Flg*^*ft/ft*^ mice. Data represent the mean and *error bars* represent ± SEMs from 6 to 8 mice and are representative of 2 separate experiments. The Student *t* test or 2-way ANOVA was used to determine statistical differences between groups. *NS*, Not significant, ∗*P* > .05. ***P < .001.

HDM allergen was applied to the intact skin of *Matt*^*ma/ma*^ and WT mice to address whether the altered skin barrier in *Matt*^*ma/ma*^ mice influences allergen sensitization. Percutaneous sensitization with allergen led to enhanced skin inflammation in *Matt*^*ma/ma*^ mice (Fig 4, *A-C*). Consistent with the defective barrier, TEWL was significantly upregulated (*P* < .001) in HDM-treated *Matt*^*ma/ma*^ mice after allergen application (Fig 4, *D*). Crucially, HDM-specific IgE responses were significantly (*P* < .05) evoked in the sera of HDM-treated *Matt*^*ma/ma*^ mice, as well as HDM-specific IgG_1_ and IgG_2a_ responses (Fig 4, *E*). In contrast, the skin of WT (Fig 4) and *Flg*^*ft/ft*^ (data not shown) mice was refractory to cutaneous HDM exposure. Collectively, these data demonstrate that *Matt*^*ma/ma*^ mice have spontaneous AD and a defective skin barrier that facilitates percutaneous allergic sensitization.

### A mutation in *MATT* is associated with AD

**Fig 5:**
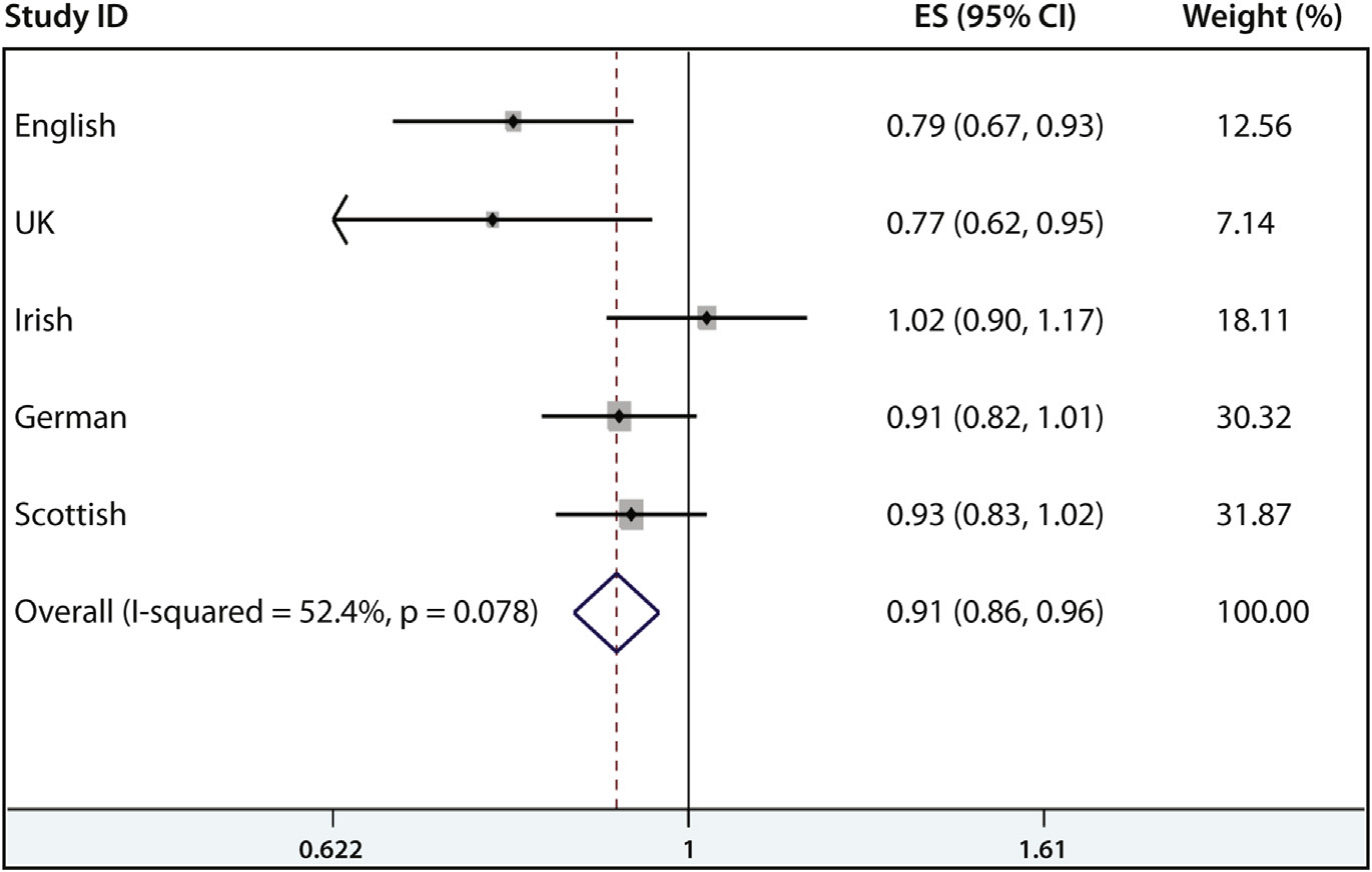
Forrest plot showing results of a fixed-effects meta-analysis of 5 case-control studies to investigate the association of rs6684514 and AD. Study populations used were as follows: *English*, English adult severe AD versus English population control subjects from the 1958 Birth Cohort; *UK*, UK mild-moderate pediatric AD versus English pediatric control subjects without AD; *Irish*, Irish pediatric AD versus Irish adult population controls; *German*, German AD cases versus German population control subjects; *Scottish*, Scottish asthma cases with AD versus Scottish population control subjects. *ES*, Estimated odds ratio. Meta-analysis was carried out with the “metan” function in Stata software (StataCorp).

To investigate whether *MATT* is associated with human AD, all exons of human *MATT* were sequenced in 55 Irish AD cases who were negative for *FLG* null mutations. Several noncoding SNPs and 1 known missense SNP, rs6684514, were identified (see Table E2 in this article's Online Repository at www.jacionline.org). Case-control analyses conducted on 2 independent AD case collections, English adult AD and United Kingdom pediatric AD, with separate population-matched control subjects (see Table E3) showed a significant association between rs6684514 and AD (Table I). The minor allele (A) is protective for disease in both the English adult AD (OR, approximately 0.791; *P* = .0038) and UK pediatric AD (OR, approximately 0.770; *P* = .0143) populations (Table I). Notably, when controlling for the strong and significant *FLG* null mutations, it was shown that the association of rs6684514 with AD is independent of *FLG* (Table I and see Table E4 in this article's Online Repository at www.jacionline.org). The association of rs6684514 with AD was further replicated in a German AD case-control analysis in which the OR after controlling for *FLG* mutations was 0.86 (95% CI, 0.76-0.97; *P* = .0161; Table I). However, this association was not replicated in Irish or Scottish case-control analyses (Table I). A meta-analysis using a fixed-effects model of all 4,245 AD cases from England, Scotland, Ireland, and Germany with 10,558 population-matched control subjects did not show significant heterogeneity between the study groups (*P* = .078). A small but significant effect of rs6684514 on AD risk was confirmed in the meta-analysis (OR, 0.91; 95% CI, 0.86-0.96; *P* = .001; Fig 5; see Table E4). A random-effects meta-analysis also produced similar results (OR, 0.90; 95% CI, 0.82-0.98; *P* = .015; see Fig E8 in this article's Online Repository at www.jacionline.org).

## Discussion

Previously, we demonstrated that *FLG* loss-of-function mutations have a very strong relevance for the common inflammatory skin disease AD and associated atopic phenotypes^3,9,10^ and identified an analogous mutation in the murine homolog *Flg* in the spontaneously occurring flaky tail DM (*maft*) mouse.^12^ Subsequently, these DM mice have been widely used as a model of heritable skin barrier deficiency and spontaneous dermatitis^13-15,17^ and as a model of filaggrin deficiency–associated AD pathogenesis in patients.^29^ In this study we have identified the *matted* phenotype in the DM mouse as arising from a second mutation in *Matt* (*Tmem79*). The *Matt*^*ma*^ mutation results in defective expression of the transmembrane protein mattrin, which is highly expressed in the upper granular layer of epidermal keratinocytes, with a predicted role in lipid homeostasis. These studies have elucidated the relative contributions of the *Flg* and *Matt* mutations in the DM mouse. The *Matt*^*ma*^ mutation led to the striking development of spontaneous AD-like skin pathology and atopy in adult mice. These data indicate that the DM mouse is not a true model of filaggrin deficiency–associated AD-like skin inflammation. It is notable that the *Flg*^*ft*^ mutation has a neonatal influence, whereas the *Matt*^*ma*^ mutation has a progressive influence with age. This indicates the polygenic nature of the DM mouse as an AD model and indicates the differential influence of both the *Flg*^*ft*^ and *Matt*^*ma*^ mutations. Recently, *Flg*^*−/−*^ mice were shown to have no overt dermatitis with age,^30^ with the authors unable to sensitize *Flg*^*−/−*^ mice by means of application of OVA, an allergen commonly used in models of skin inflammation,^31^ to the intact skin barrier, results similar to our data. Notably, *Flg*^*ft/ft*^ mice were not sensitized to HDM (data not shown), indicating an impermissibility of the *Flg*^*ft/ft*^ skin barrier to protein antigen ingress. These data indicate that the DM mouse is not a true model of filaggrin deficiency–associated AD-like skin inflammation.

In this study we adopted a translational approach and also addressed whether mattrin was implicated in human AD. A missense SNP in *MATT* was shown to have a small but significant effect on the risk for human AD. Because mattrin shows distant sequence homology to MAPEG family members,^24^ which are known to catalyze glutathione-dependent transformations of lipophilic substrates at the lipid bilayer, sequence homology suggests a possible role for mattrin in the biology of lipids or lipid-like molecules.^25^ Epidermal transcriptomic analysis of WT versus *Matt*^*ma/ma*^ mice has revealed changes in several transcripts involved in fatty acid and lipid biology (data not shown). However, 1-dimensional and 2-dimensional thin-layer chromatography of epidermal lipid extracts did not reveal any differences in the migration or abundance of ceramides, phospholipids, or polar or nonpolar lipids between WT and *Matt*^*ma/ma*^ mice (data not shown). Future work will require analysis of the glutathione binding of mattrin, elucidation of lipophilic substrates of mattrin, and an investigation of the downstream signaling pathways and local immunologic milieu in *Matt*^*ma*^ mice.

In summary, using a translational mouse-patient strategy, we have identified a new gene mutation that leads to dermatitis in mice and have further demonstrated that a variant in the human gene is associated with AD.

**Clinical implications**

The role of *MATT* mutations in susceptibility to AD and related allergic conditions should be further investigated.

## Figures and Tables

**Table I:** Results of case-control analyses to investigate the association of rs6684514 and AD in 5 populations

Case-control comparison	Cases (n)	Control subjects (n)	*P* value for rs6684514	OR	95% CI	*P* value for rs6684514 association after controlling for *FLG* null mutations
English adult severe AD vs English population control subjects from the 1958 Birth Cohort	505	1919	.0038	0.791	0.674-0.929	.0008
UK mild-moderate pediatric AD vs English pediatric control subjects without AD	338	538	.0153	0.770	0.622-0.953	.0328
Irish pediatric AD vs Irish adult population control subjects	724	1905	.1300	1.025	0.896-1.172	.7905
German AD cases vs German population control subjects	1543	2005	.0794	0.912	0.822-1.011	.0161
Scottish asthma cases with AD vs Scottish population control subjects	1135	4189	.1365	0.922	0.831-1.023	NA

*English adult severe AD* is defined as early-onset, persistent, and severe disease. *UK mild-moderate pediatric AD* includes cases collected from an English population birth cohort (n = 177) and a Scottish General Practice collection (n = 161); the *English pediatric control subjects* were ascertained not to have AD at the age of 7 to 9 years. *Irish pediatric AD* cases were collected in secondary and tertiary care clinics in Ireland; *Irish population control subjects* are healthy adult blood donors. *Scottish asthma cases with AD* are subjects with physician-diagnosed asthma and parent-reported AD. The German case and control rs6684514 genotypes were ascertained by imputation from genome-wide SNP analysis data; all other SNP genotypes and *FLG* null mutations were analyzed by using TaqMan allelic discrimination assays. In the English, United Kingdom, and Irish collections, 4 prevalent *FLG* null mutations were analyzed (R501X, 2282del4, R2447X, and S3247X), whereas in the German population 2 *FLG* null mutations were analyzed (R501X and 2282del4). Statistical analysis was performed through logistic regression in Stata 10.0 software (StataCorp).

*NA*, *FLG* genotype data not available.

## Supplementary data

Methods

Tables EI-EIV

Fig E3

Fig E8

Figs E1-E8 Legends
