# Correction

**DOI:** 10.1016/j.jaci.2015.10.002

**Published:** 2015-12

With regard to the article in the November 2013 issue entitled “*Tmem79/Matt* is the matted mouse gene and is a predisposing gene for atopic dermatitis in human subjects” (J Allergy Clin Immunol 2013;132:1121-9), the authors report an error on page 1122 in the Results section of the Abstract, in the sentence, “Meta-analysis of 4,245 AD cases and 10,558 population-matched control subjects showed that a missense SNP, rs6694514, in the human MATT gene has a small but significant association with AD.” The SNP number “rs6694514” should read “rs6684514.” The authors regret the error.

