# Supplementary material for: Tmem79/Matt is the matted mouse gene and is a predisposing gene for atopic dermatitis in human subjects
Source: J Allergy Clin Immunol. 2013 Nov;132(5):1121–9. doi: 10.1016/j.jaci.2013.08.046 (PMC3834151; doi:10.1016/j.jaci.2013.08.046)
Supplement: Methods [file mmc1.docx]

**ONLINE REPOSITORY**

**METHODS**

**Next Generation Sequencing and Bioinformatics**

The raw fastq data provided had between 67 M – 105 M 76 bp paired-end reads per replicate. Fastqc (v0.10.0 http://www.bioinformatics.bbsrc.ac.uk/projects/fastqc/) was used to check their read quality before alignment to the Ensembl 66 mouse genome ([1](#_ENREF_1)) with the BWA short read aligner ([2](#_ENREF_2)) (v0.5.9-r16 setting fastq format to Illumina v1.3+, parameter ‘-I’) ). Read counts were assigned to genes with HTSeq­count:

<http://www.huber.embl.de/users/anders/HTSeq/doc/count.html> using the following parameters: mode=union; type=exon; idattr=gene_id.

The Ensembl 66 GTF file was used for gene annotations, resulting in 28,007 genes expressed in at least one of the replicates. Differential gene expression between the WT and the Matted samples was performed with DESeq (v1.6.1) ([3](#_ENREF_3)) and R (v2.14.0) with significant genes defined as those with p<0.01. In order to concentrate on genes with the most reliable read counts, genes that had <120 reads in total (i.e. on average <20 reads per sample) across all six samples were removed, leaving 18,078 genes.

**SNP and InDel Detection Methodology**

All replicates were combined into one BAM file per sample and the aligned reads were passed through SAMtools v0.1.18 ([4](#_ENREF_4)) for calling SNPs and InDels with the standard command: samtools mpileup -C50 -DS –uf <ref> <bams> | bcftools view -bvcg > raw.bcf. The called SNPs/InDels were filtered to report only those with a minimum read depth of 20. SNPs/InDels were compared between the WT and *Matt^ma/ma^* samples and only those found in the *Matt^ma/ma^* samples were retained for further analysis. 383 SNPs and InDels were identified in the EDC region (chromosome 3: 87,000,000-95,000,000) as being unique to the *Matt^ma/ma^* sample.

**AD case collections**

The following collections of cases were used:

(1) **English adult severe AD** is early onset, persistent and severe disease, diagnosed by experienced dermatologists in secondary and tertiary care.

(2) **UK mild-moderate pediatric AD** includes cases collected from an English population birth cohort (n=177) and a Scottish Primary Care collection (n=161). AD is defined using the UK refinement of the Hanifin and Rajka diagnostic criteria.

(3) **Irish pediatric AD** cases were collected in secondary and tertiary care clinics in Ireland, defined by experienced dermatologists.

(4) **German AD** cases are adults and children diagnosed by experienced dermatologists and pediatricians using standard criteria for AD, in tertiary care clinics.

(5) **Scottish asthma cases with AD** are individuals with physician-diagnosed asthma in primary and secondary care, with parent- or self-reported history of ever having had AD.

The following matched control populations were used:

(1) **1958 Birth Cohort** comprises unselected English population controls.

(2) **English pediatric controls** were ascertained not to have AD at the age of 7-9 years. They are the hyper-normal controls from the same English population birth cohort from which the English mild-moderate pediatric AD cases were recruited.

(3) **Irish population controls** are healthy adult blood donors in Dublin, Ireland.

(4) **German population controls** include pediatric and adult cohorts from Germany.

(5) **Scottish population controls** are healthy adult blood donors from throughout Scotland.

**REFERENCES FOR ONLINE METHODS**

E1. Flicek P, Amode MR, Barrell D, Beal K, Brent S, Carvalho-Silva D, et al. Ensembl 2012. Nucleic Acids Res. 2012;40(Database issue):D84-90. Epub 2011/11/17.

E2. Li H, Durbin R. Fast and accurate short read alignment with Burrows-Wheeler transform. Bioinformatics. 2009;25(14):1754-60. Epub 2009/05/20.

E3. Anders S, Huber W. Differential expression analysis for sequence count data. Genome Biol. 2010;11(10):R106. Epub 2010/10/29.

E4. Li H, Handsaker B, Wysoker A, Fennell T, Ruan J, Homer N, et al. The Sequence Alignment/Map format and SAMtools. Bioinformatics. 2009;25(16):2078-9. Epub 2009/06/10.
