# Supplementary material for: Tmem79/Matt is the matted mouse gene and is a predisposing gene for atopic dermatitis in human subjects
Source: J Allergy Clin Immunol. 2013 Nov;132(5):1121–9. doi: 10.1016/j.jaci.2013.08.046 (PMC3834151; doi:10.1016/j.jaci.2013.08.046)
Supplement: Tables EI-EIV [file mmc2.docx]

**Table EIa**

**Primers for mouse SNP detection and positional candidate gene sequencing**

| Gene/mRNA | Location on mouse Chr3 |  | F primer (5'-3') | R primer (5'-3') |
| --- | --- | --- | --- | --- |
| 4930564K09rik | 82680531:82747696 | exon 1 | GAAAGGGACTTGGAGTGG | TAGTGTATTAGATGTGGCTCAG |
|  |  | exon 2 | CATAGAACAGGATAGTAGCACAG | CAGTGCAAAGCTGTGGTT |
|  |  | exon 3 | GATGCATGCATACTCCTAAGG | GCATCTTCCACTTCAAGGG |
|  |  |  | GCAGACCTATTGCCGTTT | GATCCTAGTTCCTTCTCCTCAC |
|  |  |  | CAGATATACATGGGATTTAAGG | TGGATGAGACTGCCATTC |
|  |  |  | CAGCTATACTATATGTCCTGAA | GGATGATGAGGGCATAGAA |
|  |  |  | CTGTGACTCTAGTTCCAAGGG | CAGTCAGCAGGTTGTTAAGG |
|  |  |  | GCTACCTGGAACACAATGA | TAGGACCATATCCATTAGAC |
| 4933430H15rik | 87540562:87552864 | exon 1 | GCGAAATGGTAAAGATGGG | GGATTTTTTGGCTCGTCC |
|  |  | exon 2-3 | GCAAAGCAGCTTCCACTTG | AGGCCAAAGTCATCTCAGG |
|  |  | exon 4-5 | CTCACGGGCTCACAATTCA | TCCCTGTGATTCCTACAGAGA |
|  |  | exon 6-7 | CTATCCTTAGGCTCAGAT | AGAACAGAGTGACTTGTAAG |
|  |  | exon 8 | GCTTACAAGTCACTCTGTTCT | CAGCAGATTTTGTTGAACTA |
|  |  | exon 9 | CAAAGTGAGCCATTTCCCTGT | CCAGAGCCCAGCTTCTCTT |
|  |  | exon 10 | CATTATAGCTGTCCCCATGT | AGGAAGACCTCCTAAGGAAG |
|  |  | exon 11 | GTTTCTCCAGCACCTGGGAC | CATCTGACATCACTGGCAGCC |
|  |  | exon 12 | GGGATGGACTTGATTCTCCAA | GTTTTAGGGCTGTTGAACGG |
|  |  | exon 13 | GTTTGTTTCCTGCAATTAG | CAAACACATGGAGATAGC |
|  |  |  | GGAACCCAGCCTTCTCTGA | CCGGTGTGTAGACCTTCTCAG |
|  |  |  | CTGGGCCAGAGCCAGTTGT | GGGAGGTCTCCCAGATTCCA |
|  |  |  | GACACTGAGAAGGCCTAGG | TCAAATGTAGCCAGCGTC |
|  |  |  | CTGTGGGAAGATGGTGAGG | CCCCCAGAACCTTCTATTTATG |
| AA008399 | 87703360:87703930 |  | CCCACATCAACCATCAAG | CAAAACCCAGGTCCTTATG |
| AW764307 | 88516367:88517054 |  | GCACCTTTAGGCACACTTG | TGTTGGGCAGGGTTTATG |
| BB160298 | 87653139:87653787 |  | TTACCCACTCTCTCCAAGG | TTTTGAAAGTGGAGGTTCC |
| BY722912 | 93075872:93076483 |  | TGACATTGGAGAAATAGAGGTG | CCATCATTCTTGCCATTAGTAG |
| CN720784 | 87830051:87831030 |  | AGTTCCAGGATAGCCAATG | TACCGCAAAGTGCCTAAG |
|  |  |  | GGACTCCATTCACACAAGC | TGACTTAGTGTGCTGCGTG |
| *Crnn* | 92948710:92953707 | intron 1 | ATGGAGGAAGAGGCTATGC | ACAAAGGAAGACGGAAGC |
| D930015E06rik | 83730775:83844469 | exon 1 | GCTGGGCTACGATTCATTC | GCGAACCCTCACAGATTTC |
|  |  | exon 2 | GGCTGGGCATTGTATTTG | TTAGAGACAGCCGTGGAGAC |
|  |  | exon 3 | CCAGAGGGTAGAGTTACAGGTG | TCTGAACAAGAGGGAAGGC |
|  |  | exon 4 | CTATTGATGATGGAGACTTTGG | CCAAACTTAGGGCTTCTCTG |
|  |  | exon 5 | GAGAAGCCCTAAGTTTGGTC | AAAGGGCAGGAAATCTCTC |
|  |  | exon 6 | GGTCCCACATCAGAAGAGAAG | TGAGCCCATAGCCCAATAG |
|  |  | exon 7-8 | GCTGTTTGGAACTCACTGTATG | TTATGGATGAGGCAGGACC |
|  |  | exon 9-10 | TGGAGAGTCTATGGCTTTTTG | CCTGAGTGAAACTGCCAAC |
|  |  | exon 11 | CCTCCCCAAACTCTTCTTTAC | AACTGGGGTTCAGGAGAAG |
|  |  | exon 12 | GGTATTTCCTGAGTTGTATTGG | GCCACAACTGAACTAAAAGC |
|  |  | exon 13 | TTTAGTCATAGCGAGTGCG | GCACAGAGGAAGGAAACTG |
|  |  | exon 14 | CACTGATGATTGTCCAGAGG | TCTCTCACAAAAGCACAAGC |
|  |  | exon 15 | CTCATAGTTTGTGAAGTTTGGC | TGCTGGCTGAAGTGCTAAC |
|  |  | exon 16 | CATTCCTCCTCTCCTCTGAC | GCACAGTGACAAAGAAGGTG |
|  |  | exon 17 | TGTAGGATGAGGTCAAGAGTTG | CACTAAAAAGGAATGCTAAGGG |
|  |  | exon 18 | TGACTCTACCCTCCATTGG | AAATGTGTGTGTGCCTGTG |
|  |  | exon 19 | TGGCATTTTAGTCTGTGAGAG | GGGAAAGAGTCAAACAACTTC |
|  |  | exon 20 | AAGTGAAGTGTATGAAACCTGC | CCATAAGGTCCTCTTCTAAAGC |
| DY243455 | 87335657:87336293 |  | CCCTCCACATTTGTTCTATTC | CAAGGTTTCTCTTTTAGCCC |
| *Flg* | 93080173:93080723 | intron 1 | CACTGAGATTCGGGCTATG | TTCCTTAGACTCTTCCTTTGC |
| *Flg2* | 93001960:93005015 | intron 1 | TCTGGAAGGACAACTACAGG | ACGATGTCACTCCAACGAG |
| GH455320 | 88508416:88509079 |  | AGTGCTGGGAAAGAGGGTAG | TCTGGGGAACTCTGGGATAC |
| gm6570 | 87483615:87484251 |  | GGGAGGAAGCAAAGAAGAG | AGAATCACCTGGGTCAATG |
| gm9790 | 85719448:85720030 |  | AGTCAAACCATTGGCTCTC | TCTAACTAAAGCAAGCACTTCC |
| map6.26 | 92559223:92559820 |  | GGAGAGGAAAAGTATGCCTG | GATAAGCATTTGGCACTGG |
| *Rptn* | 93195844:93201987 | exon 1 | CCAACCAGAGTAGACAGTAAGG | TTATCACGAGGAAGAGAACAAC |
|  |  | exon 2 | TTTGGAGACATCCTTCGG | TTATGATGGCAGGCTTGG |
|  |  |  | TGGAACACTTGGACCGAGAC | CTCTCTGTCTGACTGTTGCC |
| S100A10 | 93357462:93366990 | exon 1 | GTTAGTCTGTTGGCAGAGTG | AAGGAGAACAGAACAGCGG |
|  |  | exon 2 | CCAATACCTGAAGTGTCTGTTG | CACCTGCAGATTGAGAGTTCTT |
|  |  | exon 3 | CCTTATGCACGTGGCTGTCA | CCTGAGAGGAGAATGCTTTCCC |
| *Tchh* | 93244475:93251622 | exon 1 | TGAAATGAAAACAGGGGG | TCATCAGTGGTAATGCCC |
|  |  | intron 1 | GGTCTTGATGGCATTGTTAG | GCTGGAGAGATGGTTTAGCC |
|  |  | exon 2 | TACAGATGGGGTTGTGAGCCAC | AGTCTCTGCTCTTCATCGC |
| *TMEM79*  *(Matt)* | 88132575:88183555 | exon 1 | TTTGGAGACACAGGCAAG | TCTCTACAGCCAAGGGTCA |
|  |  | exon 2.1 | TTCCTCCCCACAGTGTTCT | AATGGCTCCTCCGACTTAG |
|  |  | exon 2.2 | CAGAGGCACCTACTAAGTCG | AGTGGCAGGTTCAGGATAC |
|  |  | exon 3 | GGTTTCTCTTCATTCTGCTG | AAGGCAATGGATTCAACAC |
|  |  | exon 4.1 | GGTGTTTGGAGTAGTATCGGC | TTTTCCTTCTCCTTGGCG |
|  |  | exon 4.2 | ACACTTCCTTCTTGGGCAG | GCTTGGAAGGCAGTTAGTTTC |
|  |  | exon 4.3 | CAAGCCTCTCTCTGTCTTCC | TGCTCCCAGTAAGTTGTCC |

**Table Ib**

**Primers for human *MATT* gene PCR and sequencing**

| Gene/mRNA | Location on human Chr1 |  | F primer (5'-3') | R primer (5'-3') |
| --- | --- | --- | --- | --- |
| *TMEM79* | 156254070:156262234 | exon 1 | GCCTGCTGCTACCAAATCT | CTTTACTCCTATGTGATGCTCG |
|  |  | exon 2.1 | TGGACGGCATTTGATGTC | TGTAGGTCAATAGGCACGAAG |
|  |  | exon 2.2 | GACGATGCCAACCTGCTG | AGGGTAGGGTCAGAAAAGGC |
|  |  | exon 3 | TTTCCCTTCACTTGACCTG | GAAATCGGCTTCCATCTG |
|  |  | exon 4.1 | TACCAGCCATACCCACCAAC | ACGGCTGTCCACCTTTTTC |
|  |  | exon 4.2 | CAGGGAAAAAGGTGGACA | TAACATCAACCACCGCAC |

**Table E1c**

**Primers for semi-quantitative RT-PCR (mouse)**

| cDNA murine primers | Location | F primer (5' - 3') | R primer (5' - 3') |
| --- | --- | --- | --- |
| *TMEM79/Matt* | chr3:88,133,776-88,136,947 | CATCTTCTTCCCCTGTCTG | CAAGAGTGGCAGGAAAGTC |
| *Krt14* | chr11:100,068,255-100,068,804 | ACTCACTCGCTCACTTGCTCA | ATCTTGCTCTTCAGGTCCTC |

**Table EII**

**SNPs detected by sequencing in human *MATT* gene**

| Chrosome position | mRNA position | dbSNP rs# cluster id | Function | Reference allele/SNP | Protein residue | Amino Acid Position |
| --- | --- | --- | --- | --- | --- | --- |
| 1:156253031 | - | rs28372828 | 5' near gene | G/C | - | - |
| 1:156254988 | 142 | rs192228711 | 5' UTR | T/C | - | - |
| 1:156255456 | 610 | rs6684514 | missense | G/A | Val>Met | 147 |
| 1:156255747 | - | - | intron | T/C | - | - |
| 1:156255833 | - | rs2842883 | intron | G/C | - | - |
| 1:156256000 | - | rs3795728 | intron | T/C | - | - |
| 1:156261491 | 1,458 | rs6679145 | 3' UTR | G/C | - | - |

**Table EIII**

**Case definitions and demographic data for AD case collections and population-matched controls**

| **Case or control collection** | **Number of individuals** | **Names of collections** | **Phenotype definition** |
| --- | --- | --- | --- |
| **English adult severe AD cases** | 505 | ? | AD defined by experienced dermatologists; early onset persistent and severe disease in patients of white European ancestry living in or near London or Newcastle-upon-Tyne; cases recruited from secondary and tertiary care |
| **UK mild-moderate pediatric AD cases** | 338 | NCCGP and GenCEP | AD defined using UK Diagnostic criteria*; mild and moderate disease defined by the Three Item Severity score**; pediatric cases recruited from an English population birth cohort (NCCGP n=177, aged 7 to 9 years) and a Scottish Primary Care case collection (GenCEP n=161, aged 0 to 16 years) |
| **Irish pediatric AD** | 724 | NCRC-ADC | AD defined by experienced dermatologists; cases aged 0 to 16 years, recruited from secondary and tertiary care |
| **German AD cases** | 1543 | German tertiary care cases | AD defined by experienced dermatologists and pediatricians according to standard criteria^*,***^; tertiary care pediatric and adult cases recruited from University Hospitals in Germany |
| **Scottish asthma cases with AD** | 1135 | BREATHE and PAGES | Pediatric and young adult physician-diagnosed asthma cases with parent- or self-reported history of ever having had AD |
| **English 1958 Birth Cohort** | 1919 | 1958 Birth Cohort | English population birth cohort |
| **Non-AD English controls** | 538 | NCCGP | English population birth cohort for whom AD was excluded at the age of 7 to 9 years |
| **Irish adult population controls** | 1905 | Trinity Biobank Controls | Irish adult blood donors from Dublin |
| **German population controls** | 2005 | KORA, PopGen and ISAAC | Population based German pediatric (ISAAC) and adult (KORA, PopGen) cohorts |
| **Scottish population Controls** | 4189 | Generation Scotland  (GS3D) | Adult blood donors from throughout Scotland |

* Williams HC, Burney PG, Pembroke AC, Hay RJ. The U.K. Working Party’s Diagnostic Criteria for Atopic Dermatitis. III. Independent hospital validation. Br J Dermatol 1994;131: 406–16.

** Acta Derm Venereol. 1999 Sep;79(5):356-9. Scoring the severity of atopic dermatitis: three item severity score as a rough system for daily practice and as a pre-screening tool for studies. Wolkerstorfer A, de Waard van der Spek FB, Glazenburg EJ, Mulder PG, Oranje AP.

*** Rajka G. Essential Aspects of Atopic Dermatitis. Berlin: Springer-Verlag; 1989.

**Table EIV**

**Case-control analyses to investigate the association of rs6684514, FLG null mutations and AD**

| **Case/control comparison** | **cases, n** | **controls, n** | **rs6684514 and AD** | | **rs6684514, *FLG* null mutations and AD** | | |
| --- | --- | --- | --- | --- | --- | --- | --- |
|  |  |  | **OR**  **(95% CI)** | **p-value** | **rs6684514 OR**  **(95% CI)** | ***FLG* OR**  **(95% CI)** | **p-value** |
| **English adult severe AD *vs* English population controls from the 1958 Birth Cohort** | 505 | 1919 | 0.791  (0.674 to 0.929) | 0.0038 | 0.709  (0.580 to 0.868) | 3.248  (2.436 to 4.331) | 0.0008 |
| **UK mild-moderate pediatric AD *vs* English pediatric controls without AD** | 338 | 538 | 0.770  (0.622 to 0.953) | 0.0153 | 0.791  (0.637 to 0.981) | 1.751  (1.260 to 2.433) | 0.0328 |
| **Irish pediatric AD vs Irish adult population controls** | 724 | 1905 | 1.025  (0.896 to 1.172) | 0.1300 | 0.981  (0.850 to 1.132) | 4.238  (3.512 to 5.162) | 0.7905 |
| **German AD cases vs German population controls** | 1543 | 2005 | 0.912  (0.822 to 1.011) | 0.0794 | 0.860  0.761 to 0.972 | 4.891  (3.891 to 6.146) | 0.0161 |

Logistic regression analysis was performed in Stata**^®^**12.0 (StataCorp, 4905 Lakeway Drive, College Station, Texas 77845 USA).
