# Supplementary material for: Tmem79/Matt is the matted mouse gene and is a predisposing gene for atopic dermatitis in human subjects
Source: J Allergy Clin Immunol. 2013 Nov;132(5):1121–9. doi: 10.1016/j.jaci.2013.08.046 (PMC3834151; doi:10.1016/j.jaci.2013.08.046)
Supplement: Fig E3 [file mmc3.docx]

Figure E3

**Bioinformatics analysis of Tmem79/mattrin Protein**

**1. Summary**

A preliminary sequence analysis of TMEM79 (NP_115699.1 | CAI14162.1) was performed using ANNOTATOR analysis framework (Schneider et al. 2010; Ooi et al. 2009). There seems to be evidence from computational predictions and the comparison to published structures to support the following findings:

1. TMEM79/mattrin and its orthologs are distantly related to the MAPEG (Membrane Associated Proteins in Eicosanoid and Glutathione metabolism) superfamily many of whose members are involved in inflammation pathways.

2. Although sequence identity is very low, a multiple sequence alignment of MPGES1 and TMEM79 together with other canonical members of the MAPEG family shows that a large percentage of the key residues involved in glutathione binding in MPGES1 are also present in TMEM79 (see section 3).

3. Since MAPEG family members catalyze glutathione-dependent transformations of lipophilic substrates harvested from the lipid bilayer (Martinez Molina et al. 2008), experimentally establishing Glutathion binding of TMEM79 could serve as a first step to validate the above predictions and decide on whether to continue with a search for a lipophilic substrate (see section 4).

**2. General Sequence Analysis**

A segment based strategy for functional analysis was used. Aaround 30 algorithms were applied to try to distinguish non-globular segments from globular domains. The non-globular segments are often regions of low-sequence complexity or compositional bias, but may carry out important structural functions or contain signals for posttranslational modification or localization. On the other hand, conserved globular domains with a fairly stable three-dimensional structure might carry out enzymatic function or serve as an interface in protein-protein interactions or have general scaffolding roles. The results can be seen in (a) and show that TMEM79/mattrin has a long N-terminal region of low sequence complexity. No further analysis of this region was undertaken. The C-terminal half of the protein is dominated by five predicted trans-membrane segments. After this sequence motif and architecture analysis, orthologous sequences were gathered which were subjected to a similar analysis as above to look for emerging patterns. Subsequently, the analysis was extended to individual segments to not only include orthologs but also remote homologs, which were gathered with an iterative procedure.

The ortholog collection algorithm was used to find a number of orthologs and subsequent analysis of these found an enrichment for PFAM domain hits (PF01124) against the MAPEG protein family

Further evidence for membership in the MAPEG family was uncovered:

1. Inspection of the HMMER result on TMEM79 finds a sub-significant hit (E-value: 0.029) against the MAPEG domain (PF01124)

2. PSI-BLAST finds members of the MAPEG family starting from round 2.

3. The locally installed version of HHPRED (Soding et al. 2005) finds a clear hit (E-value: 9.7E-11) against the MAPEG domain (PF01124)

A number of caveats should be mentioned:

• All the well-studied MAPEG members only have four TM segments, while TMEM79 has five. The region of homology clearly only extends to the last four segments of TMEM79.

• All MAPEG members appear to only as long as the TM segments together. There are no long N-terminal unstructured regions, as found in TMEM79/mattrin.

• All known structures of MAPEG members show the formation of a homotrimer.

**3. Comparison of TMEM79 and MPGES1**

There are a number of publications that provide detailed structural information as well as results of site-directed mutagenesis for MPGES1 (Microsomal prostaglandin E synthase 1) (Jegerschöld et al. 2008), LTC4S (Leukotriene C4 synthase) (Molina et al. 2007; Ago et al. 2007) and FLAP (5-Lipoxygenase-activating protein) (Ferguson et al. 2007)with the last one lacking enzymatic activity.

A multiple sequence alignment was constructed using locally installed MAFFT (Katoh et al. 2005) and taking as input the orthologs of TMEM79, MPGES1 and the seed sequences for the MAPEG domain in PFAM (PF01124). Since the seed only contained xenopus sequences for LTC4S and FLAP, the rat and human orthologs were added respectively. A small number of MAPEG seed sequences with very long loop regions were removed.

The alignment shown in (a), above, presents alignment with only the core members shown. The first two sequences are orthologs (YP_617596.1 and ZP_05250079.1) of TMEM79 which were retained to provide some visual guidance and context. The third and fourth sequence are TMEM97 and MPGES1 respectively (they are surrounded by a red dotted box). They are followed by two sequences of LTC4 and FLAP each. The last two sequences belong to the MAPEG seed and are again left there to provide some context.

The following tables (b-d), contain assessment which was done by comparing features and individual residues mentioned in (Molina et al. 2007) among the different sequences. Mismatches between residues are classified and color-coded as explained below. The same numbers and color-codes are indicated in the multiple sequence alignment shown in (a).

**4. Summary**

In summary, there seems to be a sufficient match between key residues TMEM79 of glutathione binding to merit further experimental investigation as a MAPEG-like protein.

**(a) Multiple Sequence Alignment of TMEM79, MPGES1, LTC4 and FLAP and additional members of the MAPEG family**

**
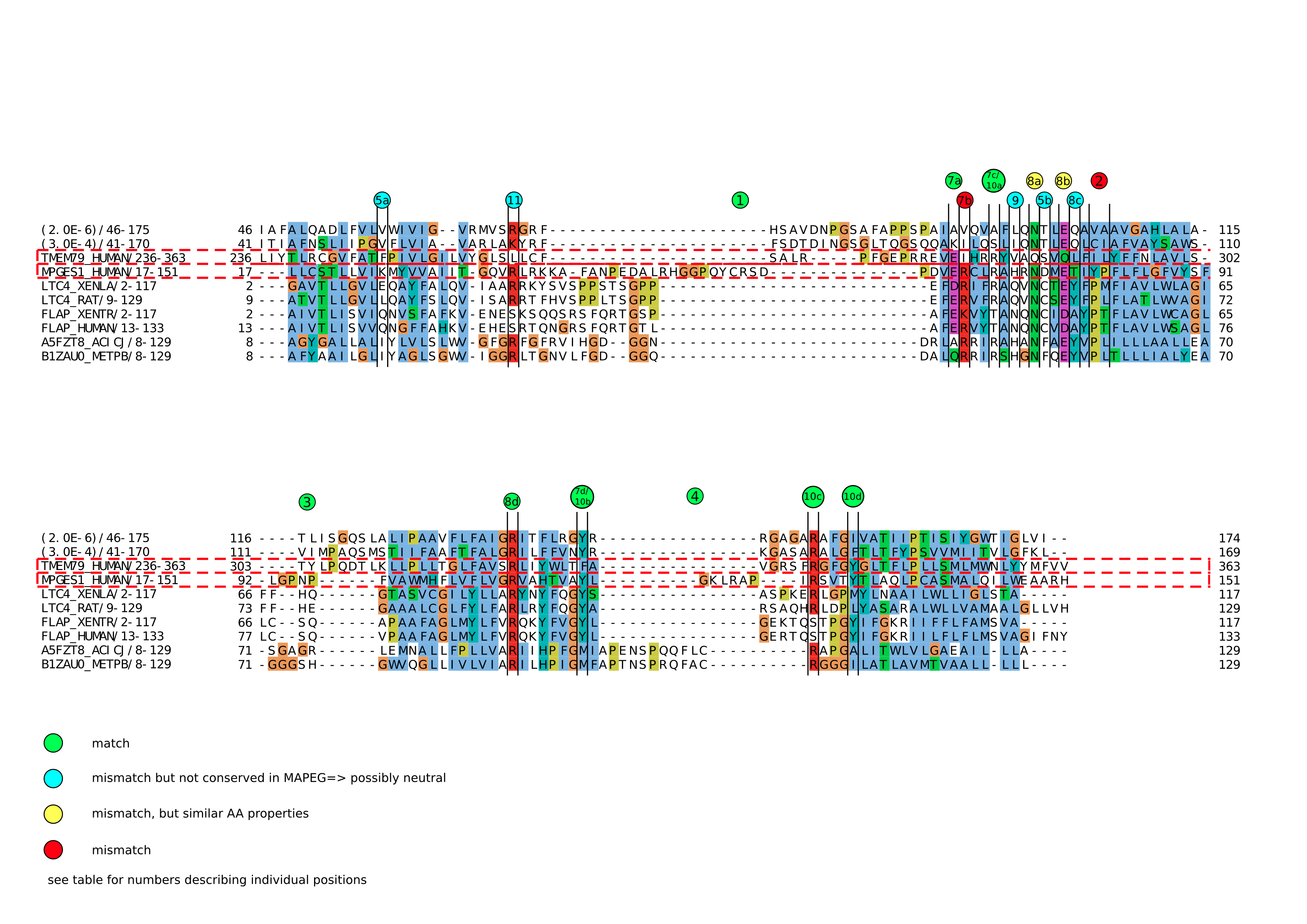
**

Key residues flagged here are detailed in b-d, below.

**(b) Evidence for possible glutathione binding**


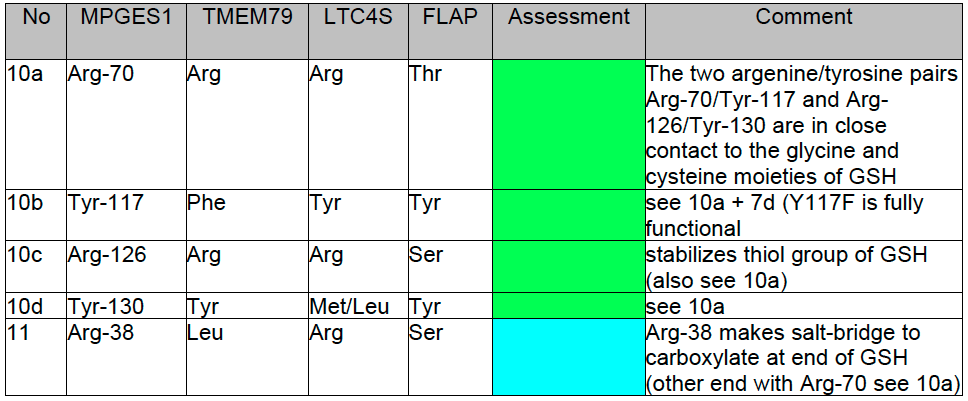


**(c) Overall structure**

**
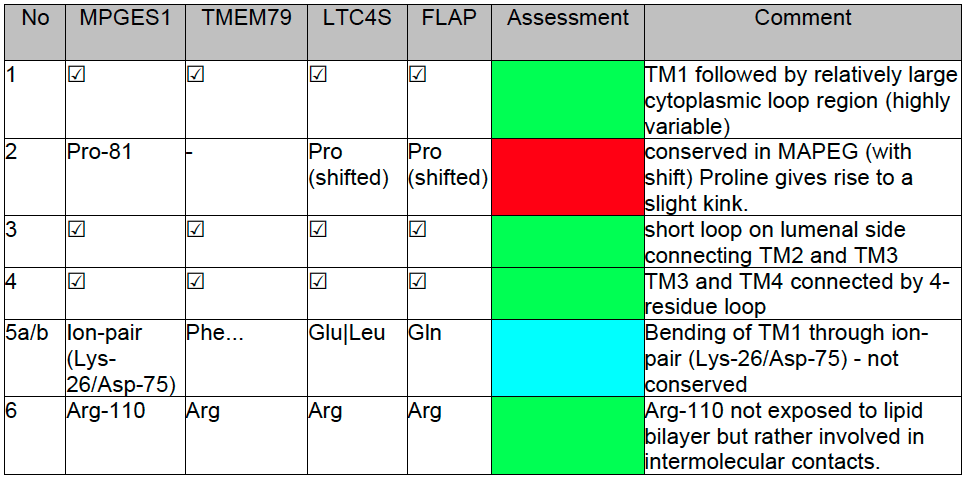
**

**(d) Results of mutagenesis experiments on known MAPEG members**

**
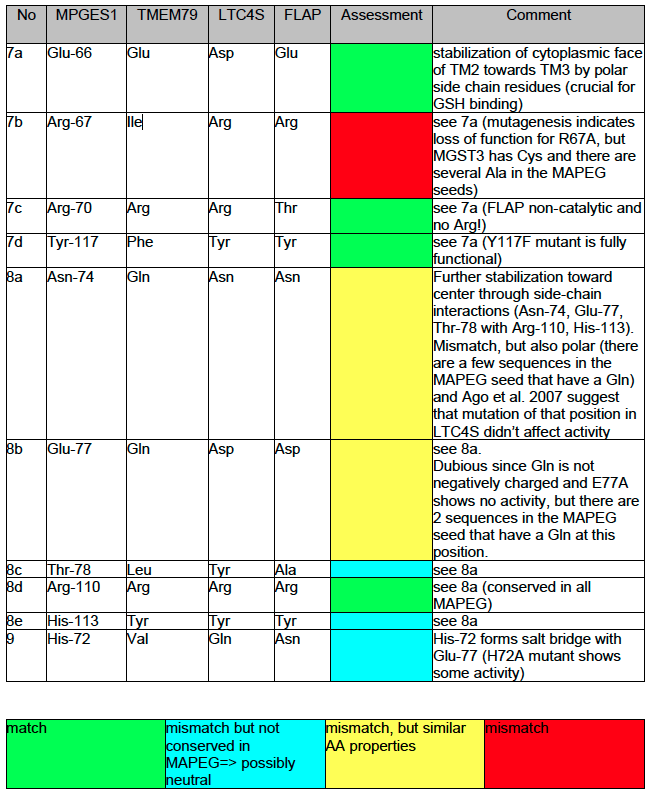
**

**5. Bibliography**

Ago, H. et al., 2007. Crystal structure of a human membrane protein involved in cysteinyl leukotriene biosynthesis. Nature, 448(7153), pp.609-612.

Ferguson, A.D. et al., 2007. Crystal structure of inhibitor-bound human 5-lipoxygenase-activating protein. Science (New York, N.Y.), 317(5837), pp.510-512.

Jegerschöld, C. et al., 2008. Structural basis for induced formation of the inflammatory mediator prostaglandin E2. Proceedings of the National Academy of Sciences, 105(32), pp.11110 -11115.

Katoh, K. et al., 2005. MAFFT version 5: improvement in accuracy of multiple sequence alignment. Nucleic acids research, 33(2), pp.511–518.

Martinez Molina, D., Eshaghi, S. & Nordlund, Pär, 2008. Catalysis within the lipid bilayer-structure and mechanism of the MAPEG family of integral membrane proteins. Current Opinion in Structural Biology, 18(4), pp.442-449.

Molina, D.M. et al., 2007. Structural basis for synthesis of inflammatory mediators by human leukotriene C4 synthase. Nature, 448(7153) pp.613-616.

Ooi, H.S. et al., 2009. ANNIE: integrated de novo protein sequence annotation. 37 (Web Server issue), p.W435-W440.

Schneider, G. et al., 2010. Integrated tools for biomolecular sequence-based function prediction as exemplified by the ANNOTATOR software environment. Methods in Molecular Biology (Clifton, N.J.), 609, pp.257-267.

Soding, J., Biegert, A. & Lupas, A.N., 2005. The HHpred interactive server for protein homology detection and structure prediction. Nucleic Acids Research, 33(Web Server), p.W244-W248.
