# Supplementary figures and images for: Tmem79/Matt is the matted mouse gene and is a predisposing gene for atopic dermatitis in human subjects
Source: J Allergy Clin Immunol. 2013 Nov;132(5):1121–9. doi: 10.1016/j.jaci.2013.08.046 (PMC3834151; doi:10.1016/j.jaci.2013.08.046)

Supplementary Figure 8

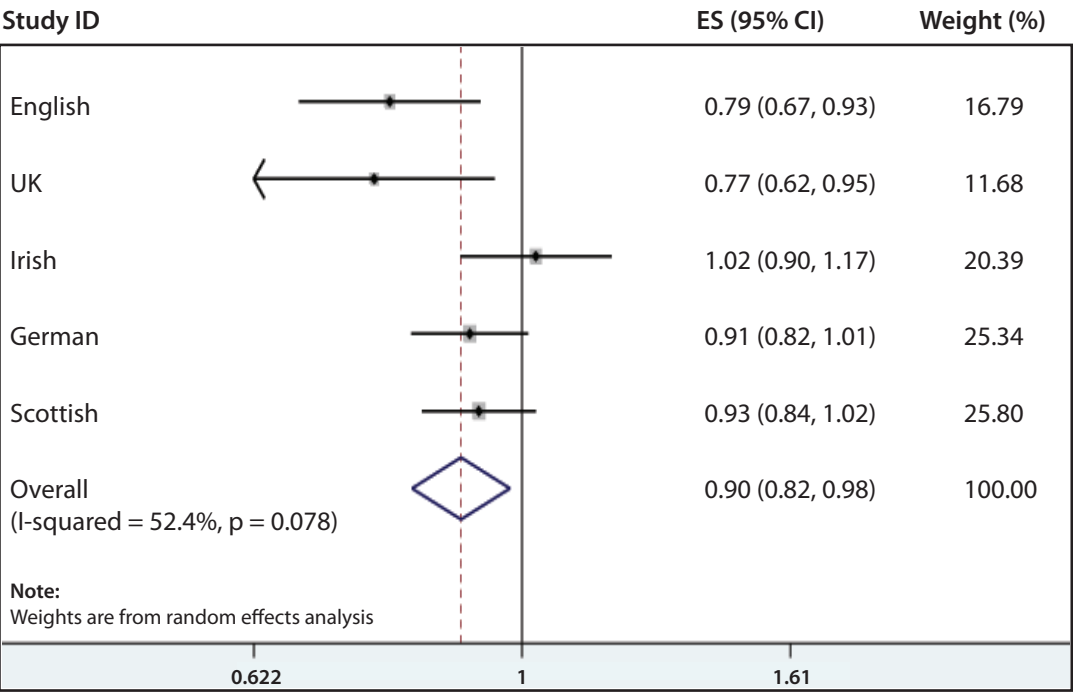

Supplement: Fig E8 [file mmc4.pdf]
