# Supplementary material for: Tmem79/Matt is the matted mouse gene and is a predisposing gene for atopic dermatitis in human subjects
Source: J Allergy Clin Immunol. 2013 Nov;132(5):1121–9. doi: 10.1016/j.jaci.2013.08.046 (PMC3834151; doi:10.1016/j.jaci.2013.08.046)
Supplement: Figs E1-E8 Legends [file mmc5.docx]

**ONLINE FIGURE LEGENDS**

**Figure E1** *Breeding strategy for generating homozygous* Matt^ma/ma^ *and* Flg^ft/ft^ *mice.*

**Figure E2** *Random recombination in chromosomal regions around the ma mutation with backcrossing, and* CviQI *restriction digest* ma *genotying assay.*

(a) Backcrossing and intercrossing strategies employed to generate congenic *Matt^ma/ma^* mice on a C57BL6/J background, resulted in random recombination each generation, gradually replacing the chromosomal regions around the *ma* causative mutation with C57BL/6J genome, resulting in the homozygous critical interval carrying the mutation to be flanked by two uneven heterozygous genomic regions. (b) A genotyping assay was developed using *CviQ* I restriction digestion. The 644 bp PCR fragment derived from WT animals contains two *CviQ* I sites, one of which is knocked out by the mutation. In this restriction fragment length polymorphism assay, WT mice yield fragments of 380, 186 and 78 bp; bands of 458 and 186 bp are present in congenic *Matt^ma/^*^ma^, JAX *Matt^ma/^*^ma^, and DM mice.

**Figure E3** *Mattrin sequence homology.*

Bioinformatics analysis of the weak sequence homology between mattrin and members of the MAPEG family of proteins.

**Figure E4** *Immunofluoresent staining for mattrin in human biopsies, and* TMEM79/MATT *expression in human tissue.*

Immunofluorescence staining of human epidermis from scalp (a) and cheek (b), shows strong expression of mattrin (green) within the granular layer of the epidermis. In (c) longitudinal and (d) transverse cryosections of human hair follicles, immunofluorescence staining revealed that mattrin is located in the inner root sheath, internal to outer root sheath marker keratin K17 (red). (e) Screening a cDNA panel of 48 human tissues by QRT-PCR revealed that *TMEM79/MATT* is highly expressed in the skin, prostate and cervix, all of which are tissues with a high component of stratified squamous epithelium.

**Figure E5** Matt^ma^ *mice develop AD-like inflammation, elevated TEWL and atopy.*

(a) Macroscopic clinical scoring. Data represents the mean; error bars represent ±SEM, from 25-30 mice per strain, scored longitudinally. (b) DM, *Matt^ma/ma^* and *Flg^ft/ft^* mice have elevated dermal cell infiltration, acanthosis and hyperkeratosis, relative to WT mice, with DM and *Matt^ma/ma^* mice having increased dermal cell infiltration, acanthosis and hyperkeratosis relative to *Flg^ft/ft^* mice. Cell numbers were counted, and acanthosis and hyperkeratosis scored, on 15-20 HPF (x1,000) on hematoxylin and eosin-stained sections from WT, DM, *Matt^ma/ma^* and *Flg^ft/ft^* mice. (c) Elevated TEWL readings in DM and *Matt^ma/ma^* mice in comparison to WT and *Flg^ft/ft^* mice. (d) Total-IgE in the serum is elevated in DM, *Matt^ma/ma^* and *Flg^ft/ft^* mice, relative to WT controls. Data represent the mean and error bars represent ±SEM (a,b,c) or min and max (c,d). Student’s t-test or two-way ANOVA was used to determine statistical differences between groups. NS- *P* > 0.05 * *P* < 0.05, *** *P* < 0.001.

**Figure E6** *Individual clinical score parameters, and severe AD pathology in* Matt^ma^ *mice.*

(a) Clinical scoring assessed by the parameters of pruritus, erythema, edema, erosion and scaling. Data represent the mean; error bars represent ±SEM, from 25-30 mice scored longitudinally. (b) Severe pathology is evident in individual DM and *Matt^ma/ma^* mice by 32 weeks, with some mice exhibiting profound lesions and excoriation, particularly on the neck and ventral flank, with occasional incidence of blepharitis and ocular edema*.*

**Figure E7** *Hair fiber and bulb morphology.*

(a) Scanning electron microscopy analysis of hair fibers from *Matt^ma/ma^* and DM mice shows defective cuticular morphology, with fragile hair fibers prone to splitting and breakage. In contrast, hair fibers from WT and *Flg^ft/ft^* mice show regular cuticle morphology. Scale bar, 20 μm. (b) Representative photomicrographs shows that the hair follicle bulbs (arrows) are at different depths within the subcutis of *Matt^ma/ma^* mice at 9-days during morphogenesis, compared to heterozygous *Matt^ma/+^* littermates, leading to hair follicle misorientation. Scale bar, 100 μm.

**Figure E8** *Forrest plot showing results of random effects meta-analysis of five case-control studies to investigate the association of rs6684514 and AD.*

Study populations used were: English, English adult severe AD vs English population controls from the 1958 Birth Cohort; UK; UK, UK mild-moderate pediatric AD vs English pediatric controls without AD; Irish, Irish pediatric AD vs Irish adult population controls; German, German AD cases vs German population controls; Scottish; Scottish asthma cases with AD vs Scottish population controls. ES = estimated odds ratio; CI = confidence interval. Meta-analysis carried out using the “metan” function in Stata® (StataCorp, College Station, Texas).
